# Framing the Exit: Pollsters, Public Opinion, and the Politics of Military Withdrawal

**DOI:** 10.1093/poq/nfaf020

**Published:** 2025-05-29

**Authors:** Daniel Silverman, Caitlan Fealing

**Affiliations:** Assistant Professor, Political Science, Carnegie Mellon Institute for Strategy and Technology (CMIST), Carnegie Mellon University, Pittsburgh, PA, US; Doctoral Student, William S. Dietrich Fellow, Carnegie Mellon Institute for Strategy and Technology (CMIST), Carnegie Mellon University, Pittsburgh, PA, US

## Abstract

What shapes public support for withdrawal from an ongoing military intervention? While there is a vast literature on the public’s support for new military interventions and its approval of interventions that are underway, there is very little research on public opinion around the explicit question of ending a military campaign on foreign soil and “going home.” This is surprising, given the salience of questions about terminating military interventions in contemporary world politics, from Afghanistan to Ukraine and beyond. In this research note, we argue that the public’s expressed appetite for exiting from an intervention is influenced in crucial ways by framing choices made by public opinion pollsters. In particular, we contend that whether pollsters frame withdrawal as an enemy victory or not and the alternate response options they provide around it can strongly impact its appeal. To test these ideas, we pair an observational analysis of public opinion polls over time on American support for military withdrawals from 1946 to 2021 with an original survey experiment conducted about the 2021 NATO withdrawal from Afghanistan. The results reveal that both the enemy victory frame and what we call the middle ground frame exert a powerful influence on public support for withdrawal, and do so across people with different partisan and foreign policy predispositions. These results help provide insight into when people support going home in war, while also extending the considerable literature on framing choices in opinion polling in new ways.

## Introduction

Does the design of survey questions systematically shape our understanding of the public’s degree of support for withdrawal from military interventions? If so, how? There is a vast literature on the public’s support for *launching* new military interventions (e.g., Jentleson 1992; Perla 2011; Brownlee 2020), but there is relatively little research on mass support for *ending* them. Meanwhile, there is extensive research about how question wording and framing choices impact the expression of political attitudes (e.g., Rasinski 1989; Borrelli and Lockerbie 2008; Huber and Paris 2013). Yet scholars have not looked at the design of survey questions about military withdrawal, even though this issue is highly salient in contemporary global politics, from Afghanistan to Gaza to Ukraine—and there is wide variation in what people are asked about it (Gallup 2005).

We argue that two framing choices in survey questions about possible military withdrawals are particularly powerful. First, building on ideas about the importance of relative gains in politics, we argue that questions that use an “enemy victory frame”—in which withdrawal from a military intervention is linked to a reviled adversary “winning”—are likely to greatly diminish the public’s appetite for leaving. Second, building on ideas about the attractiveness of safe middle options, we maintain that framing a policy toward a military intervention as more extreme by changing the set of choices that surround it will decrease its appeal. Specifically, we contend that including a “troop reduction option” will undercut the public’s support for a full military withdrawal, and that a stay-the-course option will be more attractive to citizens when it is accompanied by the more extreme idea of “doubling down.”

We test these ideas with two analyses.^1^ First, we collected the top-line results of all polls in the Roper Center for Public Opinion Research Archive in which Americans were asked about their support for withdrawing from an ongoing intervention. This represents, to our knowledge, the first analysis of polls about popular support for withdrawal over time, with 234 items from 1946 to 2021. We examine question features as well as situational variables in this dataset, finding that the enemy victory and middle ground framings strongly influence aggregate support for withdrawal. Second, we conducted an original preregistered^2^ survey experiment to further explore these effects, both to boost confidence in their causal validity and to probe their heterogeneity. The experimental results align well with our observational analysis, confirming that the framing choices in question strongly influence aggregate public support for military withdrawal—and do so across partisan and foreign policy predispositions.

These results have serious implications. Most notably, they highlight the role of pollsters’ decisions in influencing the level of expressed support for states’ foreign security policies such as military withdrawals. While there are many ingredients in state decision-making about terminating a military intervention, the degree of popular support for withdrawal is often one important input. To the extent that pollsters can shape and manipulate that support by affecting the considerations and choices people see when they voice their views, this represents a potential threat to democratic accountability on major foreign policy issues. This is of particular relevance in the United States, where an elite versus public gap has long persisted about American military interventionism and its democratic credentials (Page and Bouton 2008)—although the results resonate in a number of other societies that have engaged in domestically controversial military interventions in recent years as well, from Israel and Russia to Turkey and Iran. Scholars and practitioners should be aware of the power of these subtle framing choices by unelected elites polling their societies and vigilant to their ability to distort our picture of support for war and its termination.

## Framing Choices and Support for Withdrawal

This study extends the literature on survey question framing to engage with the salient issue of popular support for military withdrawals. Framing in general is the process by which influential actors like politicians, cultural elites, and those in the media shape how people think about an issue by altering how it is presented to them (e.g., Benford and Snow 2000; Chong and Druckman 2007). Scholars have examined how the use of different frames in survey questions affects the expression of public attitudes on a variety of different issues, including welfare policy (Huber and Paris 2013), climate change (Singh and Swanson 2017), and gun restrictions (Haider-Markel and Joslyn 2001). We contend that military withdrawals are fertile ground for question framing, and that the choices and considerations given to people on surveys in these situations can be altered in powerful ways. In particular, we argue that two choices in the design of polling items about withdrawals are likely to be most influential.

First, one powerful issue that can emerge during a military withdrawal is concern about the idea of national defeat or enemy victory. Research suggests that concerns about relative gains and losses can heavily shape people’s decision-making (Henrich et al. 2001), including in the domain of international affairs (Rousseau 2002). Such concerns are particularly salient around the question of military withdrawal. Perhaps more than when a country starts a war, the idea of ending one and “going home” is linked to concerns about suffering relative losses at the hands of the adversary, being defeated in a high-stakes contest, and the thought of the reviled enemy “winning”—specters that are often used by elites seeking to sustain support for intervention. For example, US president George W. Bush used this frame during the Iraq War, as when he asked Americans in 2005 whether they would seek victory in Iraq or “withdraw and yield the future of that country to extremists.”^3^ Pundits echoed this framing, with a prominent news editor writing that “Iraq is now a central front in the war on terrorism. If we lose, the terrorists win—big.”^4^ Polling items about withdrawals have often used this frame too. Consider a Vietnam-era poll that asked: “If President (Lyndon) Johnson were to announce tomorrow that we were going to withdraw from Vietnam and let the Communists take over, would you approve or disapprove?” Unsurprisingly, only 15 percent of respondents approved. This yields:

*Hypothesis 1 (Enemy Victory Frame): Framing withdrawal from an intervention as letting the enemy win will substantially deplete public support.*

Second, research in psychology and related disciplines has shown that people are sensitive to “context effects” when choosing between alternatives: introducing a new option to an existing choice set can make people view the original alternatives very differently (Huber, Payne, and Puto 1982). One such effect is called the “compromise effect,” in which people tend to gravitate toward safe intermediate options and eschew extremes in a variety of decision-making arenas (Simonson 1989; Beauchamp et al. 2020; Kim et al. 2022). Building on this logic, the extent to which an option is presented as intermediate versus extreme—which can be altered based on the choices around it—is likely to shape its appeal. Surveys about military withdrawal provide wide latitude in this regard. Most obviously, pollsters can ask people about their support for staying the course versus withdrawing from a war. But they can also include a scaling back or troop reduction option, which is “between” staying the course and leaving, and thus likely to be perceived as a reasonable compromise (while making withdrawal appear extreme). Likewise, they can include a “doubling down” option, which entails making *a greater* military commitment to pursue victory more aggressively. This is likely to appear extreme and make staying the course seem like the reasonable middle ground. Examples of all of these choice sets in questions about withdrawal can be found—there is a true *tabula rasa* available. For instance, a 2010 poll asked: “Do you think the US is doing the right thing by fighting the war in Afghanistan now, or should the US not be involved in Afghanistan right now?” In contrast, another in 2021 asked: “Regarding the United States role in Afghanistan, do you think the US should have withdrawn all of its troops, withdrawn some, but left troops in the country, not withdrawn any troops at all, or not withdrawn and sent more troops?” This yields:

*Hypothesis 2 (Middle Ground Frame): People will be less likely to support a policy option toward a potential withdrawal that is presented as falling at the extremes of their available choices, and more likely to choose such a policy if it is presented as falling in the middle of their available choices.*

## Polling on Withdrawal over Time

As a first step to investigate these dynamics, we collected information on all public opinion polls with at least one question that asked Americans about their attitudes toward withdrawal from a military intervention in the Roper Center for Public Opinion Research Archive’s iPoll Databank (iPoll Databank n.d.). To be included, each question had to (1) ask about a full exit of US forces from an ongoing military intervention rather than simply a reduction in troops or change in posture, (2) ask about withdrawing from an active war and not from bases with a more permanent peacetime status, and (3) ask about overall support for withdrawal, not just a narrow aspect of it like whether a proposed timetable was appropriate.

**Figure 1.**
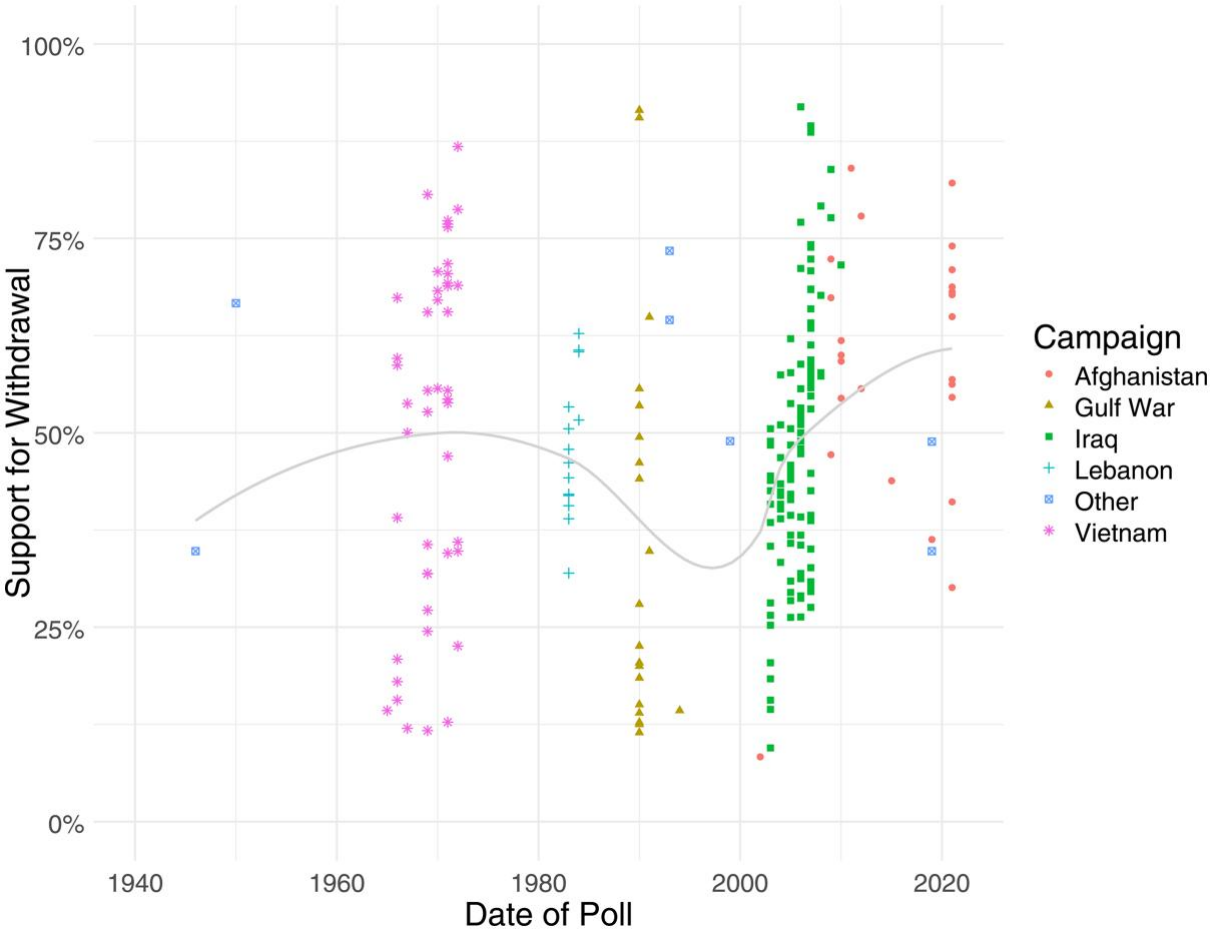
US public support for withdrawal over time. Compilation of polls from 1946 to 2021.

This produced 234 survey questions spanning 1946–2021. Figure 1 plots the top-line support for withdrawal in these polls over time. Three types of variation are evident. First, there is variation across campaigns. Roughly half the public on average wanted the United States to withdraw from Vietnam, a relatively high number consistent with the war’s divisiveness (Mandelbaum 1982; Gartner et al. 1997). Support for withdrawal falls toward 40 percent for the ensuing Lebanon and especially Gulf War campaigns, following the limited US losses in these wars. It then rises noticeably in the 2003–2011 Iraq War and especially Afghanistan, peaking at roughly 60 percent in the 2010s. This is consistent with the broad opposition to the post-9/11 wars that emerged across American society.

**Figure 2.**
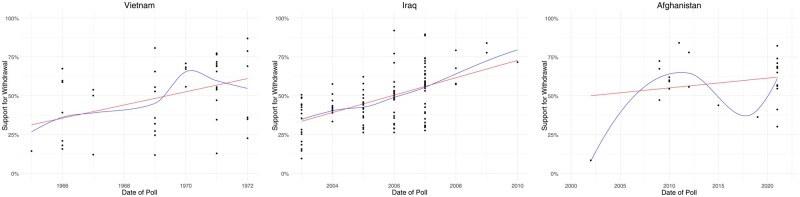
Support for withdrawal over time within Vietnam, Iraq, and Afghanistan wars.

Second, there is variation within campaigns across time. Within each major cloud of points, support for withdrawal ticks up during the latter stages of the intervention. This is consistent with literature on how early government-led narratives are upended by realities on the ground over time (Baum and Groeling 2010). This variation is highlighted more clearly in figure 2, which shows the rise in average support for withdrawal over time within the three most prominent campaigns in the dataset: those in Vietnam (left panel), Iraq (middle), and Afghanistan (right). The red line in each panel shows the linear trend within each campaign, while the blue line shows the smoothed trend within that campaign over time.

Third, there is variation between polls on the same war at around the same time. This is the most striking variation, with support varying by at least 30 percentage points in each campaign at similar time points, and more in key cases. For example, in the Gulf War, support for withdrawal varies dramatically from about 10 percent to about 90 percent during the same narrow time band. This suggests that variation in not just the reality on the ground and its coverage but in survey design may play a powerful role.

To more thoroughly investigate these data, we model the variation in aggregate support for withdrawal with both key features of the questions and contextual variables. Four question features are included. The first is a dummy for whether the question included an *Enemy Victory Framing* (H1). The second is a dummy variable for whether the question included a *Troop Reduction Option* (H2). Third, we include a dummy for whether the item was a *One-Sided Question*. Questions with a one-sided proposal for withdrawal should elicit more withdrawal support than those that present competing ideas, following the well-studied phenomenon of acquiescence bias (e.g., Keeter 2005, p. 17). Finally, we add the number of *Response Options* in each question so other key variables like *Troop Reduction Option* do not simply capture the effect of providing more choices.

We also add several contextual variables. One of these is each campaign’s *Log Cumulative Casualties* up through the month before the poll was conducted, which is expected to boost support for withdrawal (Mueller 1973). Another is *Foreign Policy Restraint*, a dummy for whether the war was aimed at stopping aggression or not, as literature argues that wars seeking to contain foreign aggression are more popular than those seeking regime change or other objectives (Jentleson 1992). Finally, we include dummy variables for each major campaign in the dataset: *Vietnam*, the *Gulf War, Iraq*, and *Afghanistan*, with other wars as the baseline. Note that we pool observations across all of the campaigns in order to maintain a sufficient sample size for analysis, reflecting the fact that we have 10 predictors and only 234 observations unevenly split across a number of campaigns. Future work could aim to model all of the campaigns separately or interact our frames with campaign-level dummies, especially if additional data could be obtained.

Table 1 shows the results. M1 includes just the question features, M2 the contextual factors, and M3 both together. *Enemy Victory Framing* and *Troop Reduction Option* both have a strongly significant influence in the expected negative direction. Their effects are substantively large too—the enemy victory frame decreases support for withdrawal by 26 percentage points, while the troop reduction option does so by 24 percentage points. This offers preliminary support for H1 and H2.

**Table 1.** Predictors of US public support for withdrawal, 1946–2021.

	(M1)	(M2)	(M3)
*Question features*			
Enemy victory framing	−26.346		−25.744
	(0.000)		(0.000)
Troop reduction option	−23.984		−23.643
	(0.000)		(0.000)
One-sided question	8.382		8.731
	(0.001)		(0.001)
No. of response options	3.297		2.708
	(0.001)		(0.003)
*Situation features*			
Log cumulative casualties		4.078	3.360
		(0.000)	(0.000)
Foreign policy restraint		−14.427	−12.183
		(0.209)	(0.208)
Vietnam		−22.482	−15.600
		(0.003)	(0.015)
Gulf War		8.939	7.222
		(0.460)	(0.479)
Afghanistan		−0.849	3.347
		(0.885)	(0.501)
Iraq		−11.555	−4.935
		(0.023)	(0.252)
Constant	41.081	30.473	24.092
	(0.000)	(0.000)	(0.000)

Observations	233	233	233
R^2^	0.277	0.143	0.404

*Note*: Results from OLS regressions. *p*-value shown in parentheses below coefficient. Coefficients are unstandardized.

## A Framing Experiment on Withdrawal Support

To dig deeper, we fielded a preregistered nationally representative survey experiment on Americans in the spring of 2023. This allows us to randomize the use of different frames to more confidently estimate their causal effects and explore their heterogeneity.

The baseline experimental question text was “Which of these comes closest to your opinion regarding the withdrawal of US forces from Afghanistan in 2021?” and the answer options always included both withdrawal and staying the course.^5^ Two aspects of the question were randomized. The first was whether the withdrawal response included an enemy victory framing, with language noting it would “let the Taliban win.” This was expected to undercut support for withdrawal (H1). The second was that the third answer choice was randomly varied between either a troop reduction option, which made staying the course seem like it was on the interventionist extreme, or a double-down option—which made staying the course seem like it was the reasonable middle ground. The expectation here was that including a double-down option would *boost* support for the status quo versus including a troop reduction option (H2).

Data were collected on Lucid from February 27 to March 4, 2023, and 1,501 responses were obtained. Participants were assigned randomly to different versions of the treatment question, yielding a relatively even distribution.^6^ In addition to the experiment, they were also asked a variety of other attitudinal and demographic questions. The dependent variable (DV) for the enemy victory hypothesis (H1) is a binary measure of support for withdrawal, coded one if the option to withdraw was chosen and zero otherwise. The DV for the middle ground hypothesis (H2) is a binary measure of support for staying the course, coded one if the option to stay the course was selected and zero otherwise. We use logistic regression models with unweighted data.

**Figure 3.**
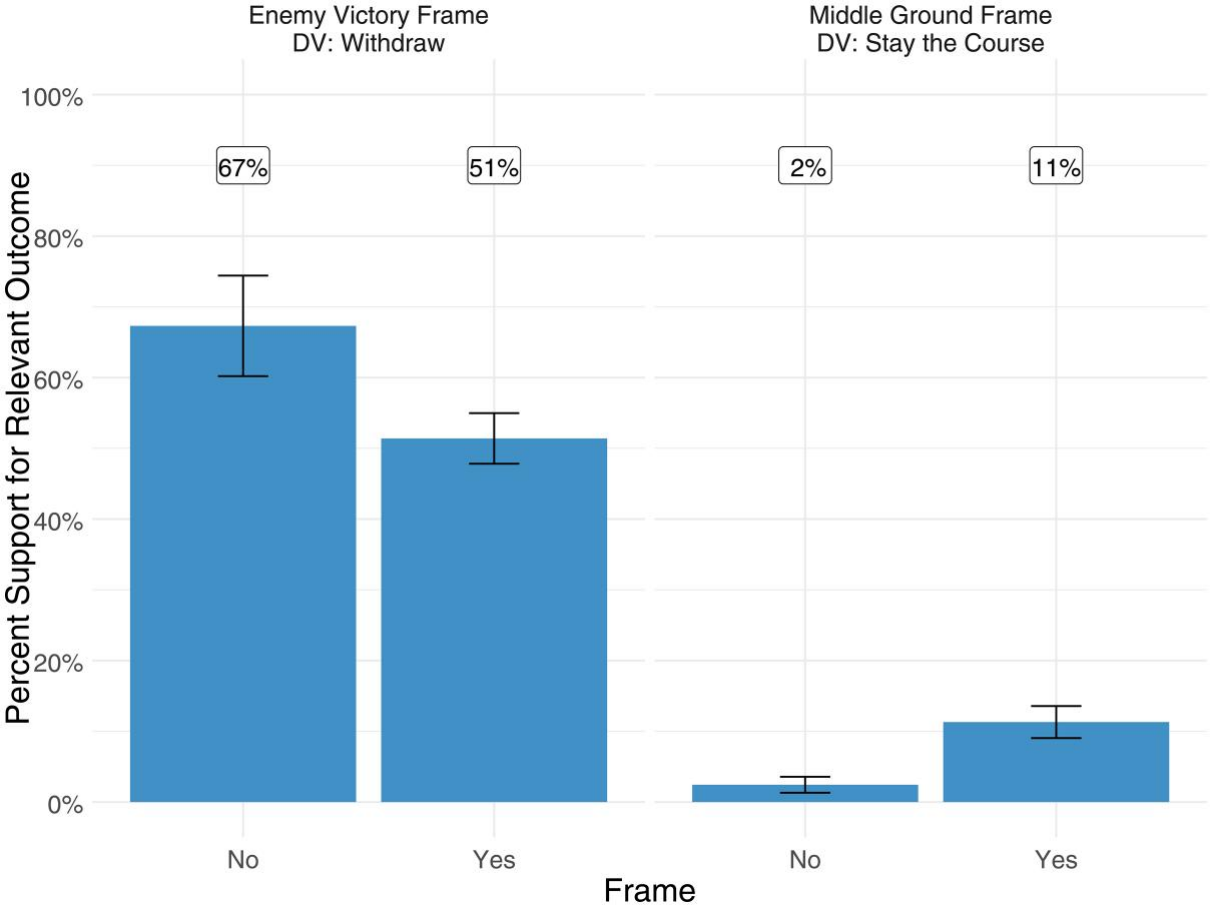
Attitudes toward withdrawal across treatment conditions.

Table 2 shows that both frames have strongly significant effects in the expected directions—the enemy victory frame significantly undercuts popular support for withdrawal, and the middle ground frame significantly boosts support for staying the course. Figure 3 shows that these effects are substantively powerful: the enemy victory frame cuts support for withdrawal from Afghanistan by 16 percentage points (from 67 percent to 51 percent), and the middle ground framing boosts support for staying the course by nine percentage points (from 2 percent to 11 percent). These results offer strong support for H1 and H2.

**Table 2.** Effects of framing on attitudes toward withdrawal.

	Dependent variable:	
	Withdraw	Stay the course
Enemy victory frame	−0.666	
	(0.000)	
Middle ground frame		1.629
		(0.000)
Constant	0.056	−2.060
	(0.444)	(0.000)

Observations	1,501	1,501

*Note*: Results from logistic regressions. *p*-value shown in parentheses below coefficient. Coefficients are unstandardized.

We also preregistered several hypotheses about treatment heterogeneity, expecting to find that the frames were more effective on some groups than others. In fact, we find little meaningful differences in their effects across people with different partisan and foreign policy predispositions (see Supplementary Material figures S1–S3), underscoring their generally potent impact.

Additional analyses and supporting information are in the Supplementary Material. These include (1) an illustration of how support for withdrawal deviates from lack of support for war in the observational data (Supplementary Material section 2), (2) a number of robustness checks for the observational analysis (Supplementary Material section 3), (3) the full wordings for the different versions of the treatment question (Supplementary Material section 4), (4) an analysis of the interaction between the two treatments on each DV (Supplementary Material section 4), and (5) a wealth of supporting information for both the data collection of the original survey (Supplementary Material section 5) and the observational analysis (Supplementary Material section 6).

## Conclusion

We studied how question design shapes support for withdrawal from military interventions. We argued that the framing choices about a withdrawal made by pollsters—specifically, whether it is framed as an enemy victory or not, and the alternative policy options surrounding it—strongly shape its appeal. We examined these ideas with both an observational analysis of aggregate support for withdrawal in US opinion polls over time and a preregistered survey experiment about US withdrawal from Afghanistan in 2021. The results offer strong support for our hypotheses, showing how the enemy victory and middle ground frames move support for withdrawal by as much as 26 percentage points in our observational analysis and 16 percentage points in our survey experiment. We also found that these effects cut across people’s ideological and foreign policy predispositions.

These findings highlight the ability of pollsters to distort the expression of foreign policy opinion. Scholars have studied how various actors can exert top-down influence on foreign policy attitudes (Knecht and Weatherford 2006; Grieco et al. 2011; Golby, Feaver, and Dropp 2018). Yet, little attention has been paid to the ability of those who actually design the questions that measure foreign policy opinion to shape it. This is surprising given the influence of public opinion models in foreign policy like those of Zaller (1992), which holds that individuals answer survey questions using considerations that are immediately accessible or “at the top of the head” (1). It follows that the frames and choice sets used by pollsters may be particularly influential, as they are directly “in front of” respondents when they are voicing their views. This is troubling from a democratic theory perspective, as pollsters are therefore a key but unelected and unscrutinized link in the articulation of foreign policy attitudes. Future research might investigate the incentives and decision-making of pollsters in this domain, for example by coding their political leanings or proximity to power and investigating how that relates to questions they employ.

## Supplementary Material

nfaf020_Supplementary_Data

## Data Availability

Replication data and documentation are available at https://dataverse.harvard.edu/dataset.xhtml? persistentId=doi%3A10.7910%2FDVN%2FMX94XS (harvard.edu).

## Footnotes

1 Replication materials for this study are available at https://doi.org/10.7910/DVN/MX94XS.

2 Our preanalysis plan is available at https://aspredicted.org/bp4ii.pdf.

3 “President’s Address to the Nation,” White House, January 10, 2007, https://georgewbush-whitehouse.archives.gov/news/releases/2007/01/20070110-7.html.

4 Morton M. Kondracke, “Bush Iraq Policy, Flawed As It Is, May Still Succeed,” *Roll Call*, October 19, 2005, https://rollcall.com/2005/10/19/bush-iraq-policy-flawed-as-it-is-may-still-succeed/.

5 We selected Afghanistan as a highly salient recent case about which people were likely to have meaningful attitudes (which may have weakened our results). However, future work might wish to examine an ongoing withdrawal.

6 ANOVA tests confirm that the conditions are demographically balanced (see Supplementary Material table S6).
