# Supplementary material for: Framing the Exit: Pollsters, Public Opinion, and the Politics of Military Withdrawal
Source: Public Opin Q. 2025 May 29;89(2):445–58. doi: 10.1093/poq/nfaf020 (PMC12369938; doi:10.1093/poq/nfaf020)
Supplement: nfaf020_Supplementary_Data [file nfaf020_supplementary_data.pdf]

Supplementary Material:

Framing the Exit: Pollsters, Public Opinion, and the Politics of Military Withdrawal

Daniel Silverman
Assistant Professor of Political Science
Carnegie Mellon Institute for Strategy and Technology (CMIST)
Carnegie Mellon University
dmsilver@andrew.cmu.edu

Caitlan Fealing
Doctoral Student
William S. Dietrich Fellow
Carnegie Mellon Institute for Strategy and Technology (CMIST)
Carnegie Mellon University
cfealing@andrew.cmu.edu

Table of Contents

Section 1: Analyses of Treatment Effect Heterogeneity... 2

Section 2: Comparison of Support for Withdrawal with Lack of Support for War...................... 5

Section 3: Robustness Checks for Observational Results 7

Section 4: Additional Analyses for Survey Experiment 15

Section 5: Additional Supporting Information on Original Survey 18

Section 6: Additional Supporting Information for Observational Analysis 19

Section 1: Analyses of Treatment Effect Heterogeneity

This section examines the heterogeneity of our two framing treatments by people's partisan and foreign policy predispositions. These moderators were all asked pre-treatment. These analyses are preregistered in our PAP (H3a-c). First, we test the expectation that the enemy victory frame will exert a stronger effect among liberals (H3a). We do this by interacting enemy victory with a standard seven-point measure of respondents' self-reported political ideology. Figure S1 presents the results. Contrary to our prediction, the enemy victory frame has a roughly constant negative and significant influence across the plot, with very little change across the ideological spectrum. This means that the frame is about evenly effective in diminishing withdrawal support among both liberals and conservatives. This result highlights the treatment's broad potency across ideology.

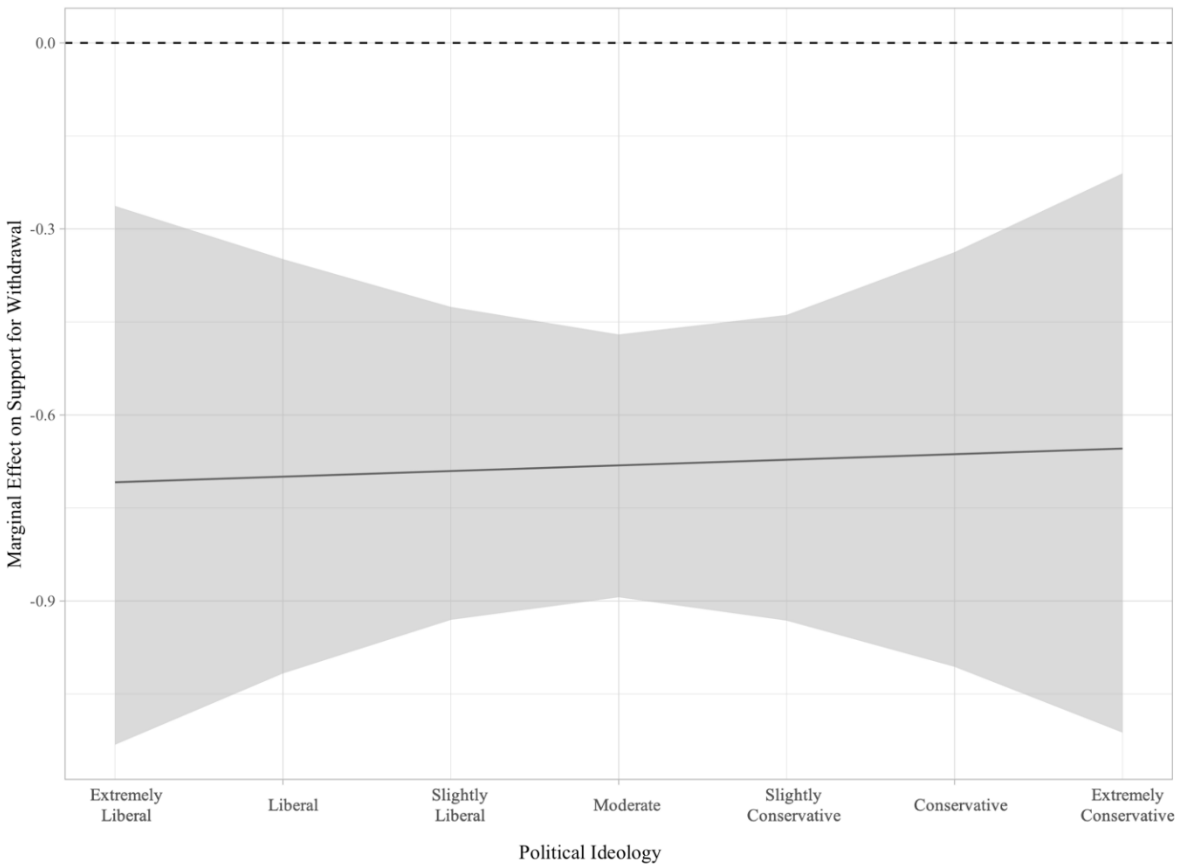

Figure S1: Interaction of Enemy Victory Frame and Political Ideology

Second, we test the expectation that the enemy victory frame will be more powerful among those with less interventionist foreign policy predispositions (H3b). Specifically, we interact the enemy victory frame with two different questions that get at people's prior willingness to support interventions like the U.S. involvement in Afghanistan. One of these is people's level of agreement with the idea that military force has a role to play in world affairs, while the other is their agreement with the idea that great powers like the U.S. and China need to play an active role in solving global problems. Participants with greater support for both of these ideas – the use of force in international affairs, and global policing by great powers – hold more interventionist predispositions and should be more supportive of U.S. involvement in Afghanistan. Following the logic of H3b, we expect that those with lower levels of these two variables will have more "room to move" and will thus be more influenced by the two treatments.

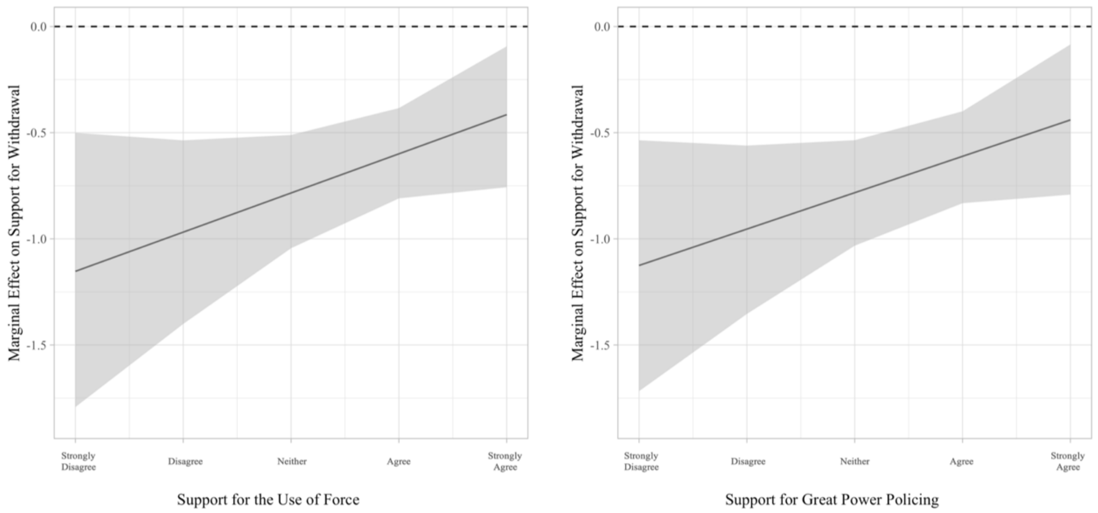

Figure S2: Interactions of Enemy Victory Frame with Foreign Policy Predispositions

Figure S2 shows the results of these tests. The left panel shows the interaction with support for military force, while the right panel shows the interaction with support for great power policing. The plots show that the impact of enemy victory is indeed more strongly negative on citizens who are less supportive of military force and great power policing. Yet, only the interaction on the right

is significant, and this only at the 10% level. Thus, the plots offer scant evidence for the idea that invoking the specter of enemy victory is stronger on dovish citizens. The clearer takeaway is that they once again highlight the broad impact of the enemy victory frame in shifting attitudes toward military withdrawal, showing that it has a statistically significant and negative influence across the spectrum of foreign policy predispositions.

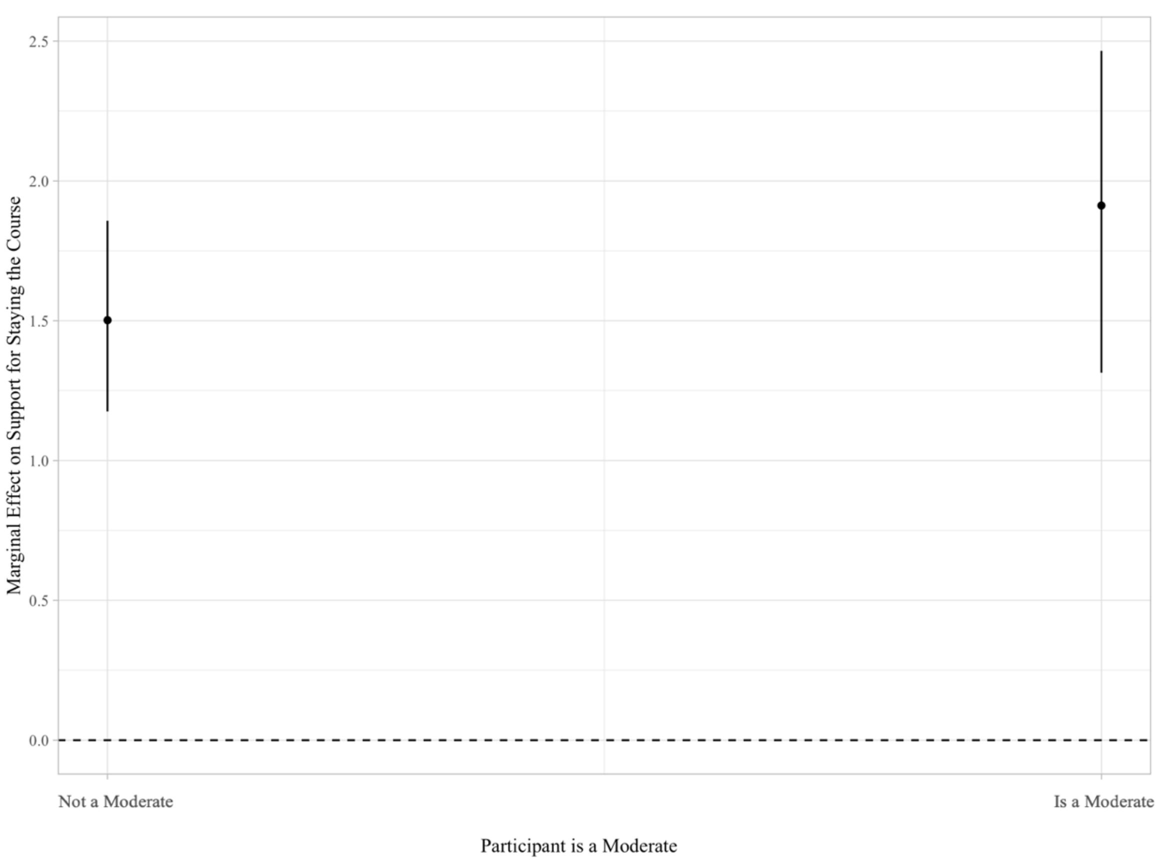

Figure S3: Interaction of Middle Ground Frame and Identifying as a Political Moderate

Finally, we also evaluate the hypothesis that self-identified political moderates will be more swayed by the middle ground treatment around the status quo option (H3c). Here we interact the middle ground treatment with a dummy variable for people who identified as “moderate, middle of the road” on the ideological spectrum. Figure S3 presents the results of this test, which shows that

the effect of the middle ground frame is indeed larger among political moderates but that this difference is not significant. In sum, our preregistered interaction tests consistently run *counter* to what we expected about the individual-level heterogeneity of the two treatment effects. Instead, they offer a potent signal that the frames in the study "work" on people with a variety of preexisting political and foreign policy orientations, as discussed in the main text.

Section 2: Comparison of Support for Withdrawal with Lack of Support for War

In this section, we compare support for withdrawal with lack of support for war in order to probe the claim that withdrawal support is an independent DV worthy of scrutiny in its own right. In order to explore this empirically, we collected all questions that asked about support for war in the Roper Database in surveys that were in our database (which necessarily include at least one question about support for withdrawal). Thus, we were able to obtain a sample of questions about support for withdrawal and support for war that come from the same polls. We chose to focus on one major campaign – the 2003-11 Iraq War – for this check since it was fairly labor-intensive.

We found 184 questions about support for the Iraq War from 91 surveys that also included at least one question about support for a military withdrawal. Figure S4 plots the toplines from the two sets of questions against each other (comparing "support for withdrawal" with "lack of support for war"). As is apparent, the two series often appear to roughly move together, although there are substantial deviations between them as well. A paired t-test of the two data series shows that there is a significant substantive *(mean diff = -9.14)* and statistical difference between them *(p = 0.000)*. This indicates that there is indeed a meaningful deviation between the two measures in a substantial segment of our data, and there are thus empirical grounds to view these two outcomes as capturing something distinct from one another.

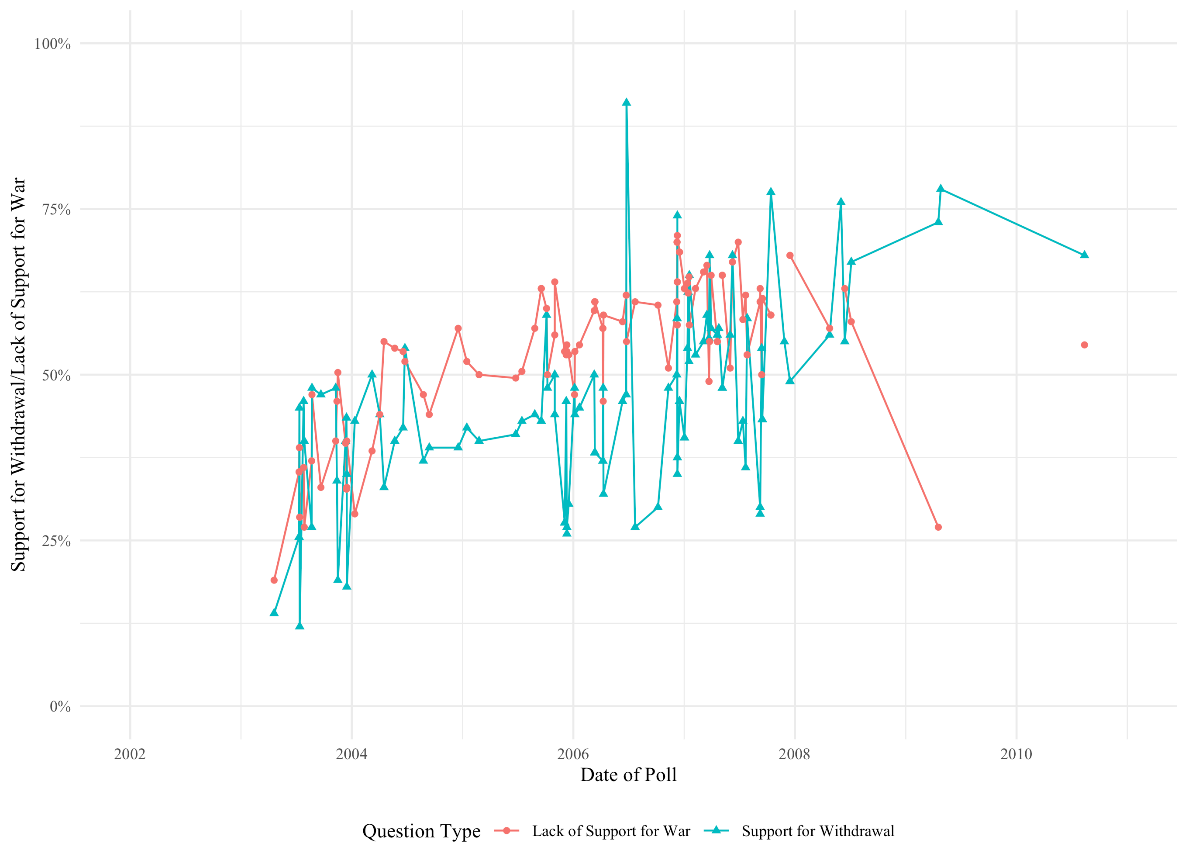

Figure S4: Support for Withdrawal vs. Lack of Support for War during the Iraq War

Section 3: Robustness Checks for Observational Results

Table S1: Replication of Observational Results with Duration of War and Logged Change in Casualties

	(1)	(2)	(3)	(4)
Context Features				
War Duration		0.042		0.083
		(0.325)		(0.098)
Log Casualties Difference			0.621	1.486
			(0.449)	(0.126)
Question Features				
Enemy Victory Framing	-25.744	-25.663	-25.795	-25.705
	(0.000)	(0.000)	(0.000)	(0.000)
Troop Reduction Option	-23.643	-23.535	-23.745	-23.670
	(0.000)	(0.000)	(0.000)	(0.000)
One-sided Question	8.731	7.956	9.124	8.126
	(0.001)	(0.003)	(0.001)	(0.002)
No. of Response Options	2.708	2.650	2.771	2.742
	(0.003)	(0.004)	(0.003)	(0.003)
Situation Features				
Log in Change Casualties	3.360	2.804	3.234	1.949
	(0.000)	(0.005)	(0.001)	(0.086)
Foreign Policy Restraint	-12.183	-10.226	-11.844	-7.472
	(0.208)	(0.301)	(0.222)	(0.455)
Vietnam	-15.600	-13.748	-17.164	-15.653
	(0.015)	(0.039)	(0.011)	(0.021)
Gulf War	7.222	4.898	6.828	1.646
	(0.479)	(0.640)	(0.504)	(0.877)
Afghanistan	3.347	-1.764	4.269	-4.634
	(0.501)	(0.806)	(0.405)	(0.532)
Iraq	-4.935	-4.336	-5.438	-4.945
	(0.252)	(0.319)	(0.213)	(0.257)
Constant	24.092	26.291	22.875	25.560
	(0.000)	(0.000)	(0.001)	(0.000)
Observations	233	233	233	233
R^2	0.404	0.406	0.405	0.413

Note: Results from OLS regressions. Table adds duration of war (in months) and logged change in casualties (prior month to current month) to base models. P-value shown in parentheses below coefficient. Coefficients are unstandardized.

Table S2: Replication of Observational Results with Dummy Variables for Number of Response Options

	(1)	(2)	(3)
Question Features			
Enemy Victory Framing	-26.641		-25.974
	(0.000)		(0.000)
Troop Reduction Option	-23.002		-22.240
	(0.000)		(0.000)
One-sided Question	6.626		7.568
	(0.009)		(0.004)
No. Response Option Dummies	Yes		Yes
Situation Features			
Log Cumulative Casualties		4.078	3.325
		(0.000)	(0.000)
Foreign Policy Restraint		-14.427	-15.895
		(0.209)	(0.103)
Vietnam		-22.482	-18.493
		(0.003)	(0.005)
Gulf War		8.939	9.126
		(0.460)	(0.369)
Afghanistan		-0.849	-0.528
		(0.885)	(0.919)
Iraq		-11.555	-8.435
		(0.023)	(0.064)
Constant	50.088	30.473	34.411
	(0.000)	(0.000)	(0.000)
Observations	233	233	233
R^2	0.327	0.143	0.433
Note:	Results from OLS regressions. Table adds dummy variables for the number of response options to base models. P-value shown in parentheses below coefficient. Coefficients are unstandardized.		

Table S3: Replication of Observational Results with SEs Clustered by Campaign

	(1)	(2)	(3)
Question Features			
Enemy Victory Framing	-26.346		-25.744
	(0.000)		(0.000)
Troop Reduction Option	-23.984		-23.643
	(0.001)		(0.001)
One-sided Question	8.382		8.731
	(0.009)		(0.117)
No. of Response Options	3.297		2.708
	(0.002)		(0.003)
Situation Features			
Log Cumulative Casualties		4.078	3.360
		(0.155)	(0.155)
Foreign Policy Restraint		-14.427	-12.183
		(0.160)	(0.132)
Vietnam		-22.482	-15.600
		(0.147)	(0.200)
Gulf War		8.939	7.222
		(0.546)	(0.554)
Afghanistan		-0.849	3.347
		(0.897)	(0.522)
Iraq		-11.555	-4.935
		(0.135)	(0.396)
Constant	41.081	30.473	24.092
	(0.000)	(0.071)	(0.080)

Note: Results from OLS regressions. P-value shown in parentheses below coefficient. Coefficients are unstandardized.

Table S4: The Effects of Each Frame in the Campaign in Which It Is Most Common – Enemy Victory in Vietnam and Troop Reduction in Iraq

	(Vietnam)	(Iraq)
Enemy Victory Framing	-33.958	
	(0.001)	
Troop Reduction Option		-21.489
		(0.000)
One-sided Question	-7.549	8.664
	(0.460)	(0.015)
No. of Response Options	-8.459	3.698
	(0.097)	(0.001)
Constant	83.633	40.319
	(0.001)	(0.000)
Observations	45	121
R^2	0.305	0.321

Note: Results from OLS regressions. P-value shown in parentheses below coefficient. Coefficients are unstandardized.

Table S5: Replication of Observational Results with Controls for Percent of Options Qualifying as Withdrawal

	(1)	(2)	(3)
Question Features			
Enemy Victory Framing	-25.836		-23.857
	(0.000)		(0.000)
Troop Reduction Option	-15.934		-14.784
	(0.000)		(0.000)
One-sided Question	7.797		8.277
	(0.001)		(0.001)
No. of Response Options	3.589		2.997
	(0.001)		(0.001)
Perc Withdraw Options	49.512		49.607
	(0.000)		(0.000)
Situation Features			
Log Cumulative Casualties		4.078	3.843
		(0.000)	(0.000)
Foreign Policy Restraint		-14.427	-16.124
		(0.209)	(0.082)
Vietnam		-22.482	-24.948
		(0.003)	(0.001)
Gulf War		8.939	8.997
		(0.460)	(0.355)
Afghanistan		-0.849	-4.883
		(0.885)	(0.332)
Iraq		-11.555	-12.413
		(0.023)	(0.005)
Constant	16.592	30.473	2.708
	(0.005)	(0.000)	(0.700)
Observations	233	233	233
R^2	0.347	0.143	0.462
Note:	Results from OLS regressions. Table adds control for percent of response options that count as withdrawal to base models. P-value shown in parentheses below coefficient. Coefficients are unstandardized.		

Table S6: Replication of Observational Results with Controls for Survey Mode and House Fixed Effects

	(1)	(2)
Question Features		
Enemy Victory Framing	-26.915	-25.993
	(0.000)	(0.000)
Troop Reduction Option	-21.755	-24.160
	(0.000)	(0.000)
One-sided Question	7.733	5.846
	(0.002)	(0.021)
Situation Features		
Log Cumulative Casualties	3.658	3.788
	(0.000)	(0.000)
Foreign Policy Restraint	-19.309	-24.756
	(0.063)	(0.019)
Vietnam	-21.779	-24.834
	(0.003)	(0.002)
Gulf War	13.813	10.383
	(0.206)	(0.315)
Afghanistan	2.390	-4.784
	(0.640)	(0.448)
Iraq	-6.196	-9.039
	(0.156)	(0.114)
Survey Method		
Method: Phone	-8.327	
	(0.060)	
Method: Web-Based Survey	-6.873	
	(0.338)	
House Fixed Effects		
CBS		-1.059
		(0.805)
Gallup		3.989
		(0.331)
ORC		-5.714
		(0.213)
Princeton		-3.812
		(0.393)
Hart		-7.842
		(0.090)
NYT		-8.184
		(0.080)
TNS		-13.676
		(0.006)
Louis Harris		4.738
		(0.412)
ABC		-9.093
		(0.341)

Washington Post		-3.906
		(0.687)
LA Times		-2.228
		(0.698)
Constant	39.062	38.615
	(0.000)	(0.000)
Observations	233	233
R^2	0.389	0.443
Note:	Results from OLS regressions. Table adds controls for survey mode as well as fixed effects for major survey firms (10+ observations). P-value shown in parentheses below coefficient. Coefficients are unstandardized.	

Table S7: Replication of Observational Results with Standard Errors Clustered by Poll

	(1)	(2)	(3)
Question Features			
Enemy Victory Framing	-26.346		-25.744
	(0.000)		(0.000)
Troop Reduction Option	-23.984		-23.643
	(0.000)		(0.000)
One-sided Question	8.382		8.731
	(0.002)		(0.004)
No. of Response Options	3.297		2.708
	(0.002)		(0.007)
Situation Features			
Log Cumulative Casualties		4.078	3.360
		(0.001)	(0.001)
Foreign Policy Restraint		-14.427	-12.183
		(0.304)	(0.311)
Vietnam		-22.482	-15.600
		(0.004)	(0.008)
Gulf War		8.939	7.222
		(0.565)	(0.575)
Afghanistan		-0.849	3.347
		(0.864)	(0.395)
Iraq		-11.555	-4.935
		(0.007)	(0.166)
Constant	41.081	30.473	24.092
	(0.000)	(0.000)	(0.000)

Note: Results from OLS regressions. Standard errors clustered by poll. P-value shown in parentheses below coefficient. Coefficients are unstandardized.

Section 4: Additional Analyses for Survey Experiment

This section contains additional analyses and supporting information related to the survey experiment. First, we present the results of covariate balance tests that were done across a number of demographic variables to check that the random assignment appears to have been successful. Then, we explore the extent to which there are meaningful interactions between the two treatments on each outcome.

Table S8: Covariate Balance Tests

Variables	***F-Value***	***Pr(>F)***
Cooperate with Different People	0.35	0.55
Trust Different People	0.03	0.86
Opinion on Use of Military Force	0.27	0.60
Opinion on Great Powers	0.14	0.71
Political Affiliation	1.32	0.57
Views of Other Cultures	0.00	0.99
Religion	0.41	0.52
Religious Importance	2.98	0.08
Gender	0.57	0.45
Age	0.06	0.80
Race	0.28	0.60
Education Level	0.74	0.39
Income	1.29	0.26
In the Military?	0.42	0.52

Note: Results are from ANOVA tests

Table S6 shows the results of covariates balance tests across 14 different individual-level variables about respondents. The tests were done with ANOVA across the four different treatment groups. As is clear, none of the variables show a level of imbalance across conditions that reaches conventional statistical significance.

We now examine the interaction between the two framing treatments – enemy victory and middle ground – that were the focus of our study. In fact, this interaction was not theorized in our PAP and intended to be included in our study, but we accidentally included a fourth condition that interacted the two frames when we ran the experiment. Table S7 thus shows the different versions of the experimental treatment question, including the fourth interactive condition (bottom right) that was accidentally added – this combines both the enemy victory and middle ground treatments. Note that this "extra" element does not impinge upon our core findings in any way, but we explore its effects here in the appendix since readers may have a substantive interest in it.

Table S9: Different Versions of Experimental Treatment Question

		Middle Ground Framing	
		No	Yes
Enemy Victory Framing	No	1. We were right to withdraw from Afghanistan. We did not belong there.<br>2. We should have reduced the number of troops we had in Afghanistan, but not fully withdrawn at that time.<br>3. We should have kept our forces in Afghanistan. They were carrying out important missions there.	1. We were right to withdraw from Afghanistan. We did not belong there.<br>2. We should have kept our forces in Afghanistan. They were carrying out important missions there.<br>3. We should have increased the number of troops we had in Afghanistan in order to strive for a complete victory.
	Yes	1. We were right to withdraw from Afghanistan and let the Taliban win. We did not belong there.<br>2. We should have reduced the number of troops we had in Afghanistan, but not fully withdrawn at that time.<br>3. We should have kept our forces in Afghanistan. They were carrying out important missions there.	1. We were right to withdraw from Afghanistan and let the Taliban win. We did not belong there.<br>2. We should have kept our forces in Afghanistan. They were carrying out important missions there.<br>3. We should have increased the number of troops we had in Afghanistan in order to strive for a complete victory.

Table S8 displays the effects of the interaction of our two treatments on both outcomes of interest. The table shows that both interactions do not yield a significant result, meaning that there is no evidence that the treatments work differently on either outcome in combination than they do individually. However, the analysis of these interactions does allow us to look at something else that was untheorized but may be of interest to readers – the effect of each of the two frames on the

"other" outcome. That is, recall that each frame was only supposed to impact one DV in our pre-registered design: the enemy victory frame was expected to diminish support for withdrawal, and the middle ground treatment was expected to boost bolster for staying the course. Here, with the inclusion of the "constitutive terms" for both interactions making it necessary, we see the impacts of middle ground on withdrawal and enemy victory on status quo as well.

The effects are meaningful and intuitive. The middle ground treatment – which in practice entails including double down as opposed to troop reduction as the third option – fuels support for withdrawal. In other words, exposing people to an extreme interventionist option not only boosts support for the status quo but also for withdrawal. Meanwhile, the enemy victory treatment boosts support for staying the course, which is also a straightforward and intuitive finding given the threat-oriented thinking it evokes. Thus, these results show that enemy victory not only undercuts support for leaving but also bolsters support for a status quo response. These results are generally consistent with those examined in the main text and help to flesh out the picture of how the two frames work to influence attitudes toward withdrawal more fully.

Table S10: Effects of Framing on Attitudes Toward Withdrawal with Interaction of Treatments

	Dependent variable:	
	Withdraw	Stay the Course
	(1)	(2)
Enemy Victory Frame	-0.506	0.456
	(0.001)	(0.052)
Middle Ground Frame	0.520	1.591
	(0.001)	(0.000)
Enemy Victory*Middle Ground	-0.325	0.100
	(0.127)	(0.721)
Constant	-0.203	-2.308
	(0.051)	(0.000)
Observations	1,501	1,501
Note:	Results from logistic regressions. P-value shown in parentheses below coefficient. Coefficients are unstandardized.	

Section 5: Additional Supporting Information on Original Survey

This section provides additional information on the data collection for our original survey. The survey was conducted through Lucid Theorem, which is a platform that aggregates participants from various online sources to build representative US opinion surveys and has become increasingly popular in political science (Coppock and McLellan 2019). Respondents on Lucid are drawn from this broader participant pool and invited to participate in individual online studies.

Of the 1,654 participants who clicked on our study, 146 chose not to participate and seven chose to exit early. Thus, data from 1,501 participants were used. These participants were assigned randomly to one of the four different versions of the experimentally-manipulated question, yielding a fairly even distribution across groups (with 376, 375, 375, and 374 individuals, respectively). In addition to receiving one of the questions about withdrawal, respondents were also asked about their political ideology, attitudes toward those who are different from them, views about other cultures, attitudes about the use of military force and the role of great powers in international affairs, and a battery of standard questions about their demographic backgrounds. The survey took approximately five minutes to complete and respondents were paid $1.50 for their participation. Besides those who chose not to participate or opted to exit early, respondents were not dropped from the analysis due to inattention, speeding, or other such criteria.

Bibliography:

Coppock, Alexander, and Oliver A. McClellan. 2019. "Validating the Demographic, Political, Psychological, and Experimental Results Obtained by a New Source of Online Survey Respondents." *Research & Politics* 6(1). https://doi.org/10.1177/2053168018822174

Section 6: Additional Supporting Information for Observational Analysis

This section provides additional supporting information about the observational analysis, specifically about the polls, non-polling data sources, and coding procedures used to generate it. The data were gathered mainly by two graduate student researchers under my close supervision. The students carefully searched through the Roper iPoll database, gathering all available polling questions about US support for military withdrawal that met the study's key criteria. As stated in the manuscript, the three primary criteria for each question to be included were that it needed to: (1) ask about the full exit of US forces from an ongoing military intervention, rather than simply a reduction in troops or change in posture, (2) ask about withdrawing from an active conflict and not from bases with a more permanent peacetime character, and (3) ask about overall support for withdrawal, not just a narrow aspect of it like whether a proposed timetable was appropriate. It should be noted that for the first criterion, questions that asked about a military withdrawal but with limited token or retainer forces staying in the country (or which were otherwise very close in substantive meaning to a full withdrawal) were still allowed.

We provide substantial supporting information on the data collection and analysis for the observational analysis beyond what is shown in the manuscript here. First, to give readers a clearer sense of data inclusion and exclusion practices, we present an illustrative list of a number of related questions that were ultimately *excluded* from the dataset because they did not meet one or more of the criteria that was required for the study – along with the reasons for their exclusion:

Table S11: Examples of Considered but Excluded Questions from Roper iPoll Database

Polling Item	Exclusion Reason
"Do you think the United States should or should not set a timetable for the withdrawal of US troops from Iraq in 2008?"	Not clear if measuring support for timetable or policy course of withdrawal more broadly
"President (Ronald) Reagan has announced that he is pulling troops out of Beirut and moving them to the ships off the coast of Lebanon. The US (United States) will then use its ships and planes from offshore to aid the Lebanese government. Do you approve or disapprove of this action?"	Withdrawal involved but simultaneously asks about supporting the Lebanese government
"Now I'm going to read you some pairs of statements. After I read each pair, please tell me whether the first statement or the second statement comes closer to your own view, even if neither is exactly right...First statement: The current course cannot bring stability and we need to start reducing the number of US (United States) troops in Iraq. Second statement: We must stay the course to achieve stability and finish the job in Iraq...(If First/Second statement, ask:) (Do you feel that way strongly or not so strongly?)"	Measures support for troop reduction, not withdrawal
"One Senator recently claimed that setting a date for a US (United States) withdrawal from Iraq is more accurately described as a date for surrender--do you think it is accurate to compare withdrawal with surrender?"	Measures whether people equate withdrawing with surrendering, not withdrawal per se
"Some say that a full withdrawal of US troops from Afghanistan would leave a vacuum that would allow terrorist groups like ISIS (Islamic militants operating in Syria and Iraq) to expand. Do you agree or disagree with this point of view? Would you say you agree strongly, agree somewhat, disagree somewhat, or disagree strongly?"	Measures whether withdrawal is equated with giving up on fighting terrorism, not withdrawal per se
"Barack Obama has decided he will delay withdrawal of US troops from Afghanistan and leave nearly ten thousand troops there through the end of 2015. Do you approve or disapprove of this new plan?"	Measures people's approval of delaying withdrawal, about timing not policy course
"(Do you think military aid to the following countries should be increased, decreased, kept the same or stopped altogether?) Afghanistan"	Measures support for military aid, not withdrawal

Additionally, we present the written coding instructions that were developed and provided to the graduate student researchers to help them capture all of the polling-related variables used in the study. These include instructions for capturing the meta-data from each polling question, the study's dependent variable (support for withdrawal), and the polling-related independent variables (question features) used in the observational analyses.

Table S12: Coding Instructions for Key Polling-Related Variables Used in the Study

Variable	Coding Instructions
Roper ID #:	*What was the ID number of the poll in the Roper system?*
Polling org:	*What was the organization that conducted the poll?*
Poll Info:	*What was the name or number of the specific poll or poll series?*
Question #:	*What was the specific question number?*
Target state/Campaign:	*In what state was the U.S. intervention?*
Start date:	*On what day, month, year did the poll start?*
End date:	*On what day, month, year did the poll end?*
N	*What was the sample size?*
Sample	*What kind of sample was it? (as reported in Roper)*
Mode	*How was the poll conducted? (as reported in Roper)*
Withdraw	*Record the percentage of the sample* <u>*supporting*</u> *withdrawal. Combine strong and weak support.*
Not withdraw	*Record the percentage of the sample* <u>*opposing*</u> *withdrawal. Combine strong and weak opposition*
DK/NR	*Record the percentage of the sample that said they didn't know or declined to respond*
Enemy victory	*Does the question specifically note that withdrawal would represent a "victory" by the adversary, such as the Taliban, Communists, Russia, Syrian regime, etc.?*
Troop reduction	*Are respondents given a troop reduction option in addition to status quo and withdrawal options, so that the former seems more like the/a "middle" choice?*
One-sided	*Is this a one-sided question? (Note that a one-sided question means that respondents are given a statement, proposal, idea, etc. and asked how much they like or support it. A two-or-more sided question means they are offered multiple competing ideas and asked which one they prefer.)*
# response ops.	*How many different response options did people have in the question, besides "don't know" or "no response"?*
# withdrawal ops.	*How many different response options were counted as expressing support for withdrawal?*

As for the contextual variables, those used in the observational analyses were primarily twofold: (1) data on military fatalities and (2) campaign objectives. Starting with the campaign objectives, these were coded by the student researchers following the work of Jentleson (1992) and his strategy of identifying the "principal policy objective" (PPO) of military interventions – in particular splitting them into internal policy change (IPC), foreign policy restraint (FPR), and humanitarian interventions. In more recent work, Eichenberg (2005) added peacekeeping which was retained in a subsequent relevant study by Brownlee (2020). The goals of all the campaigns in the dataset were coded following this typology with close reference to Jentleson (1992) – and to Brownlee (2020) for more recent campaigns. The FPR vs. IPC distinction is the principal one in this

literature and where almost all of the empirical variation in our data is, and so the models used in the study simply include an FPR dummy variable.

Meanwhile, the data on military fatalities was sourced from U.S. government estimates, specifically the information that is available on the Defense Casualty Analysis System (DCAS) platform (https://dcas.dmdc.osd.mil/dcas/app/conflictCasualties). For more recent conflicts such as the American interventions in Iraq ("Operation Iraqi Freedom") and Afghanistan ("Operation Enduring Freedom") following 9/11, we used the available summary statistics on monthly fallen soldiers in these wars provided in aggregate form on DCAS. For earlier conflicts such as WWII, Korea, and Vietnam, we used the Access to Archival Databases (AAD) tool for mining DCAS's more detailed and larger casualty records in these cases (https://aad.archives.gov/aad/index.jsp). These files were downloaded as is and then simply collapsed by month for each relevant conflict, then the two types of files were combined.

Finally, as a reference for the reader as well as for potential replication purposes, we present the full list of questions from the Roper iPoll database that were used in the observational analysis. This list is 26 pages, and so is more easily presented here at the end of the document.

Table S13: Complete List of Questions Used from Roper iPoll Database

- The United States still has several thousand troops in China. Some people say we should bring these troops home right away. Others say we should leave the troops there while General Marshall is trying to bring about peace and unity in China. What do you think?
  - Q25
  - https://doi.org/10.25940/ROPER-31095007

- It has been suggested that both the United States and communist China take their troops out of Korea and let the South Koreans and other members of the United Nations finish the war and police the country. Would you approve or disapprove of this?
  - Q6
  - https://doi.org/10.25940/ROPER-31087451

- This summer, during the rainy season in Vietnam, the Communists have said they will mount a land offensive to drive the Americans out of South Vietnam. Do you favor the U.S. (United States) sending in more troops to defend south Vietnam this summer or, do you think we should just keep the men we have there, or do you think we should take out most of the troops we have there?
  - Q23
  - https://doi.org/10.25940/ROPER-31103178

- Would you approve of continuing the fighting (in Vietnam) if it meant several hundred American soldiers would be killed every week?
  - Q23
  - https://doi.org/10.25940/ROPER-31107603

- If President (Lyndon) Johnson were to announce tomorrow that we were going to withdraw from Vietnam and let the Communists take over, would you approve or disapprove?
  - Q18
  - https://doi.org/10.25940/ROPER-31107603

- Suppose you had to choose among continuing the present situation (in Vietnam) indefinitely, fighting a major war with hundreds of thousands of casualties, or withdrawal of American troops leading to an eventual Communist takeover. Which would you choose?
  - Q21
  - https://doi.org/10.25940/ROPER-31107603

- (Suppose you had to choose among continuing the present situation (in Vietnam) indefinitely, fighting a major war with hundreds of thousands of casualties, or withdrawal of American troops leading to an eventual Communist takeover. Which would you choose?) (If continue present situation, ask:) Suppose we couldn t continue the present policy, how would you choose between a major war and a withdrawal of American troops?
  - Q22
  - https://doi.org/10.25940/ROPER-31107603

- Here is a question about U.S. policy in Vietnam. Which one of these policies do you most prefer?
  - Q9
  - https://doi.org/10.25940/ROPER-31087711

- Which would you consider the best thing that you would like to see happen in Vietnam...both sides withdraw troops under U.N. (United Nations) supervision, win total military victory over Communists, have U.S. (United States) move its troops out, or get neutralist government of South Vietnam?
  - Q21
  - https://doi.org/10.25940/ROPER-31107550

- If you have to choose, which of these courses would you choose in, Vietnam: have both sides withdraw under United Nations supervision, win a total military victory, get a neutralist government of South Vietnam, or have U.S. (United States) troops moved out?
  - Q31
  - https://doi.org/10.25940/ROPER-31107555

- If you have to choose, which of these courses would you choose in Vietnam: have both sides withdraw under United Nations supervision, win a total military victory, get a neutralist government of South Vietnam or have U.S. (United States) troops moved out?
  - Q23
  - https://doi.org/10.25940/ROPER-31107508

- If you have to choose, which of these courses would you choose in Vietnam. Have both sides withdraw under United Nations supervision, win a total military victory, get a neutralist government of South Vietnam or have U.S. (United States) troops moved out?
  - Q4
  - https://doi.org/10.25940/ROPER-31107525

- Now I want to hand you a card with four statements on it. Each statement has a different point of view about the war in Vietnam. Please read all four statements before telling me which one you agree with most. 1)I disagree with present policy. We should go further, such as carrying the ground war more into North Vietnam. 2)I agree with what we are doing but we should increase our military effort to win a clear military victory in South Vietnam. 3)I agree with what we are doing, but we should do more to bring about negotiations such as a cease fire. 4)I disagree with present policy. We shouldn t be there. We should pull our troops out now.
  - Q3
  - https://doi.org/10.25940/ROPER-31107525

- Some U.S. Senators are saying that we should withdraw all our troops from Vietnam immediately. Would you favor or oppose this?
  - Q3
  - https://doi.org/10.25940/ROPER-31087762

- On this card are a number of different approaches that have been suggested for ending the war in Vietnam. As I read each one to you, please follow along carefully. None of these may exactly fit your views, but for each one I d like to know whether you favor or oppose such a plan, and how strongly.... The United States would decide to withdraw from Vietnam, and to complete its withdrawal within the next six months. Although we would try to get whatever favorable terms are possible, we would be willing to agree to anything the other side wants in order to let us get the war over and get out.... Are you strongly in favor of this, somewhat in favor of it, somewhat opposed to it, or strongly opposed to it?
  - Q18
  - https://doi.org/10.25940/ROPER-31107624

- If North Vietnam continues to refuse reasonable proposals for peace made by the United States, there are several possible actions that could be taken. For each one, please tell me whether you would be for or against this type of action.... We should get out immediately on North Vietnam s terms
  - Q24
  - https://doi.org/10.25940/ROPER-31107624

- (If North Vietnam continues to refuse reasonable proposals for peace made by the United States, there are several possible actions that could be taken. For each one, please tell me whether you would be for or against this type of action.)... We should halt withdrawals of American troops from Vietnam until we receive some evidence that the North Vietnamese are reducing their level of activity
  - Q28
  - https://doi.org/10.25940/ROPER-31107624

- (On this card are a number of different approaches that have been suggested for ending the war in Vietnam. As I read each one to you, please follow along carefully. None of these may exactly fit your views, but for each one I d like to know whether you favor or oppose such a plan, and how strongly.)... The United States would continue its present course of action and withdraw from Vietnam over a two-year period. During that time we would continue the level of military operations about as it is now. We would continue to negotiate with the other side to obtain as many favorable terms as we could before completing the withdrawal.... Are you strongly in favor of this, somewhat in favor of it, somewhat opposed to it, or strongly opposed to it?
    - Q20
    - https://doi.org/10.25940/ROPER-31107624

- Now look at these approaches to ending the war in Vietnam again. Read each one carefully. If you had to choose one course of action from these four, which one would it be? A. The United States would decide to withdraw from Vietnam, and to complete its withdrawal within the next six months. Although we would try to get whatever favorable terms are possible, we would be willing to agree to anything the other side wants in order to let us get the war over and get out get the other side to agree to terms that are reasonably favorable to us, such as free elections in South Vietnam under international supervision. To do this we would take necessary military actions, such as blockade of the port of Haiphong and, as a last resort, selective bombing of North Vietnam. No additioU.S B. The United States would decide to end the war in Vietnam with a compromise settlement within six months. During those six months, we would try to U.S. troops would be sent. C. The United States would continue its present course of action and withdraw from Vietnam over a two-year period. During that time we would continue the level of military operations about as it is now. We would continue to negotiate with the other side to obtain as many favorable terms as we could before completing the withdrawal. military pressure, except that we would not use nuclear weapons. We would continue to fight in Vietnam until a military victory was won, and the war was ended only on terms D. The United States would decide to achieve a military victory in Vietnam. To do this, we would apply maximum completely favorable to our side. E. No opinion
    - Q22
    - https://doi.org/10.25940/ROPER-31107624

- A United States Senator has proposed legislation to require the withdrawal of all United States troops from Vietnam by the end of next year. The fighting would be turned over entirely to the South Vietnamese. With the United States providing military supplies and financial help, would you like to have Congress pass or defeat such a proposal?
    - Q4
    - https://doi.org/10.25940/ROPER-31087768

- Suppose you really knew that our presence in Vietnam for several more years would prevent Communist take-overs in other countries of Southeast Asia. Would you be willing or not willing to keep our troops there?
    - Q10
    - https://doi.org/10.25940/ROPER-31107476

- Do you favor or oppose withdrawing all of our troops from Vietnam immediately?
    - Q4
    - https://doi.org/10.25940/ROPER-31107476

- Which one of the statements listed on this card comes closest to describing the military course that you, yourself, think the United States should now pursue in the Vietnam War?
    - Q1
    - https://doi.org/10.25940/ROPER-31087772

- If the reductions of U.S. troops continued at the present rate in Vietnam and the South Vietnamese government collapsed, would you favor or oppose continuing the withdrawal of our troops?
    - Q50
    - https://doi.org/10.25940/ROPER-31107574

- If the reductions of U.S. troops continued at the present rate in Vietnam and the South Vietnamese government collapsed, would you favor or oppose continuing the withdrawal of our troops?
    - Q32
    - https://doi.org/10.25940/ROPER-31107579

- If the reduction of U.S troops continued at the present rate in Vietnam, and the South Vietnamese government collapsed, would you favor or oppose continuing the withdrawal of our troops?
    - Q33
    - https://doi.org/10.25940/ROPER-31107577

- It has been proposed that Congress pass a resolution requiring that all U.S. troops be withdrawn from Vietnam by the end of 1971. Opponents say such a resolution would tie the hands of the President. Would you favor or oppose a resolution in Congress which would require all U.S. troops be withdrawn by the end of 1971?
    - Q34
    - https://doi.org/10.25940/ROPER-31107577

- A proposal has been made in Congress to require the U.S. government to bring home all U.S. troops from Vietnam before the end of this year (1971). Would you like to have your Congressman vote for or against this proposal?
    - Q16
    - https://doi.org/10.25940/ROPER-31087797

- Suppose the choice in Vietnam came to this: either we could keep 50,000 to 100,000 U.S. troops in Vietnam for two or more years and be sure of a non-Communist government in South Vietnam, or we could bring home all American troops by the end of 1971 and have a coalition government in South Vietnam with Communists in the government. Which would you prefer -- to keep 50,000 to 100,000 U.S. troops in Vietnam or have a coaltion government in South Vietnam?
    - Q62
    - https://doi.org/10.25940/ROPER-31107586

- A proposal has been made in Congress to require the U.S. government to bring home all U.S. troops before the end of this year. Would you like to have your Congressman vote for or against this proposal?
    - Q7
    - https://doi.org/10.25940/ROPER-31107613

- With regard to Vietnam, which of these possible actions do you favor -- the immediate withdrawal of all U.S. troops from Vietnam, the withdrawal of all U.S. troops by the end of 1971, should we withdraw troops but take as long as is necessary to turn the war over to the South Vietnamese, or should we keep troops there until we win a complete military victory?
    - Q6
    - https://doi.org/10.25940/ROPER-31107613

- With regard to Vietnam, which of these possible actions do you favor -- the immediate withdrawal of all U.S. troops from Vietnam, the withdrawal of all U.S. troops by the end of 1971, should we withdraw troops but take as long as is necessary to turn the over the South Vietnamese, or should
we keep troops there until we win a complete military victory?
    - Q6
    - https://doi.org/10.25940/ROPER-31107612

- A proposal has been made in Congress to require the U.S. government to bring home (from Vietnam) all U.S. troops before the end of this year. Would you like to have your Congressman vote for or against this proposal?
    - Q6
    - https://doi.org/10.25940/ROPER-31107611

- Would you favor or oppose a Congressional act which would require all U.S. troops to be withdrawn from Vietnam by the end of 1971?

- Q57
- https://doi.org/10.25940/ROPER-31107586

- With regard to Vietnam, which of these possible actions do you favor -- the immediate withdrawal of all U.S. troops from Vietnam, the withdrawal of all U.S. troops by the end of 1971, should we withdraw troops but take as long as is necessary to turn the war over to the South Vietnamese, or should we keep troops there until we win a complete military victory?
    - Q4
    - https://doi.org/10.25940/ROPER-31107611

- Would you favor withdrawal of all U.S. troops by the end of 1971 even if it meant a Communist takeover of South Vietnam?
    - Q7
    - https://doi.org/10.25940/ROPER-31107616

- A proposal has been made in Congress to require the U.S. government to bring home all U.S. troops before the end of this year. Would you like to have your Congressman vote for or against this proposal?
    - Q6
    - https://doi.org/10.25940/ROPER-31107616

- A proposal has been made in Congress to require the U.S. government to bring home all U.S. troops before the end of this year. Would you like to have your Congressman vote for or against this proposal?
    - Q9
    - https://doi.org/10.25940/ROPER-31107617

- Would you favor withdrawal of all U.S. troops by the end of 1971 even if it threatened the lives of United States POWs (prisoner of war) held by North Vietnam?
    - Q8
    - https://doi.org/10.25940/ROPER-31107616

- Suppose one candidate for Congress from your district said that he favors getting all U.S. armed forces out of Vietnam by July 1 of next year. He is opposed by a candidate who says we must leave about 50,000 troops there to help the South Vietnamese. Other things being equal, which candidate would you prefer?
    - Q5
    - https://doi.org/10.25940/ROPER-31087804

- If the reductions of U.S. troops continued at the present rate in Vietnam and the South Vietnamese government collapsed, would you favor or oppose continuing the withdrawal of our troops?
    - Q23
    - https://doi.org/10.25940/ROPER-31107590

- A proposal has been made in Congress to require the U.S. government to bring home (from Vietnam) all U.S. troops before the end of this year. Would you like to have your Congressman vote for or against this proposal?
    - Q19
    - https://doi.org/10.25940/ROPER-31107768

- A Senate committee has voted to force the withdrawal of U.S. troops from Indochina by cutting off money after December 31st (1972), provided North Vietnam first agrees to return all American prisoners. Do you favor or oppose this?
    - Q30
    - https://doi.org/10.25940/ROPER-31087817

- Do you agree of disagree with the following positions taken by critics of the President (Nixon)?...

The President should set a deadline and order the evacuation of all American forces and let the communists take over. Do you agree or disagree with this position?

- Q14
- https://doi.org/10.25940/ROPER-31107768

• (I am going to read you some of the steps that Senator McGovern said he would take if elected President to bring an end to the war in North Vietnam, and I would like you to tell me whether you agree or disagree with each.)... End all military and political support to South Vietnam. Do you agree or disagree?
  - Q11
  - https://doi.org/10.25940/ROPER-31107777

• (I am going to read you some of the steps that Senator McGovern said he would take if elected President to bring an end to the war in North Vietnam, and I would like you to tell me whether you agree or disagree with each.)... Withdraw all American forces and all military equipment from all of Southeast Asia within 90 days. Do you agree or disagree?
  - Q12
  - https://doi.org/10.25940/ROPER-31107777

• As you may have heard, a tentative settlement of the Vietnam war has been agreed to between the U.S. and North Vietnam, including an internationally supervised cease-fire and return of the POWs (prisoners of war). I am going to read you several of the reported points of that agreement. For each one please tell me whether you approve or disapprove of it.... All U.S. bombing in Vietnam and mining of North Vietnamese harbors will stop and all U.S. forces including advisors will be withdrawn within two months. Do you approve or disapprove?
  - Q6
  - https://doi.org/10.25940/ROPER-31107781

• I m going to mention three methods of using the U.S. ships and planes stationed off the coast of Lebanon, and I d like you to tell which one method you approve of the most: a. They should be used to protect only the U.S. Marines on land when those Marines are under attack b. They should be used to defend not only the U.S. Marines, but also to help the Lebanese government fight the groups that are trying to overthrow it, including the Syrians and the PLO. c. They should not be used at all. Instead the ships and the Marines in Lebanon should be withdrawn.
  - Q50
  - https://doi.org/10.25940/ROPER-31103827

• Would you say the U.S. should send more troops to Lebanon, leave the number of troops about the same, or remove the troops that are there now?
  - Q52
  - https://doi.org/10.25940/ROPER-31103827

• Would you say the United States should send more troops to Lebanon, leave the number about the same, or remove the troops that are there now?
  - Q5
  - https://doi.org/10.25940/ROPER-31103830

• Would you say the United States should send more troops to Lebanon, leave the number about the same, or remove the troops that are there now?
  - Q6
  - https://doi.org/10.25940/ROPER-31103814

• Would you say the United States should send more troops to Lebanon, leave the number about the same, or remove the troops that are there now?
  - Q2
  - https://doi.org/10.25940/ROPER-31103813

• Would you say the United States should send more troops to Lebanon, leave the number about the same, or remove the troops that are there now?
  - Q5
  - https://doi.org/10.25940/ROPER-31103828

- Would you say the United States should send more troops to Lebanon, leave the number about the same, or remove the troops that are there now?
  - Q5
  - https://doi.org/10.25940/ROPER-31103829

- Do you think the U.S. forces should be brought back home now, or do you think they should stay and continue their peacekeeping mission?
  - Q6
  - https://doi.org/10.25940/ROPER-31103896

- Now I want to ask a few questions about the situation in Lebanon. The United States has about 1,600 marines stationed in Lebanon and another 2,000 in warships off the coast of Lebanon. Would you say the U.S. should send more troops to Lebanon, leave the number about the same, or remove the troops that are there now?
  - Q7
  - https://doi.org/10.25940/ROPER-31103831

- Would you say the U.S. should send more troops to Lebanon, leave the number about the same, or remove the troops that are there now?
  - Q19
  - https://doi.org/10.25940/ROPER-31103832

- Would you say the U.S. should send more troops to Lebanon, leave the number about the same, or remove the troops that are there now?
  - Q3
  - https://doi.org/10.25940/ROPER-31103975

- Would you say the U.S. should send more troops to Lebanon, leave the number about the same, or remove the troops that are there now?
  - Q34
  - https://doi.org/10.25940/ROPER-31086630

- Would you say the U.S. should send more troops to Lebanon, leave the number about the same, or remove the troops that are there now?
  - Q1
  - https://doi.org/10.25940/ROPER-31103972

- What do you think the United States should do next in Lebanon? Should it withdraw all U.S. Marines and Navy warships from the area, or should it continue our present policy with the Marines on ships off the coast, or should it send in a large enough military force to restore stability in Lebanon?
  - Q10
  - https://doi.org/10.25940/ROPER-31091191

- Just from what you have heard, read or seen, which of these statements comes closer to how you, yourself, feel about the United States presence in the Mideast? (Rotate)
  - Q11
  - https://doi.org/10.25940/ROPER-31088668

- Here are some conditions under which the United States might consider withdrawing its troops from the Mideast. For each, please tell me if you favor or oppose the U.S. withdrawing its troops if that condition is met.... If Iraq allows all U.S. and other foreign citizens to leave Kuwait but Iraqi troops remain in Kuwait and Saddam Hussein remains in power
  - Q13
  - https://doi.org/10.25940/ROPER-31088669

- (Here are some conditions under which the United States might consider withdrawing its troops from the Mideast. For each, please tell me if you favor or oppose the U.S. withdrawing its troops if that condition is met.)... If Iraq pulls all of its troops out of Kuwait but Saddam Hussein remains

in power in Iraq

- Q14
- https://doi.org/10.25940/ROPER-31088669

• Do you favor or oppose leaving U.S. (United States) troops in Saudi Arabia if it becomes a long, drawn-out military conflict?

- Q14
- https://doi.org/10.25940/ROPER-31088684

• Just from what you have heard, read or seen, which of these statements comes closer to how you, yourself, feel about the United States presence in the Mideast?

- Q15
- https://doi.org/10.25940/ROPER-31088685

• Do you favor or oppose leaving U.S. troops in Saudi Arabia if it becomes a long, drawn-out military conflict?

- Q7
- https://doi.org/10.25940/ROPER-31088668

• Just from what you have heard, read or seen, which of these statements comes closer to how you, yourself, feel about the United States presence in the Mideast?

- Q8
- https://doi.org/10.25940/ROPER-31088669

• (Here are some conditions under which the United States might consider withdrawing its troops from the Mideast. For each, please tell me if you favor or oppose the U.S. withdrawing its troops if that condition is met.)... If Iraq pulls all of its troops out of Kuwait and Saddam Hussein is removed from power in Iraq

- Q15
- https://doi.org/10.25940/ROPER-31088669

• Just from what you have heard, read or seen, which of these statements comes closer to how you, yourself, feel about the United States presence in the Mideast? (Rotate) A. The United States should begin to withdraw its troops or B. The United States should continue with its present level of troop presence, or C. The United States should increase the level of its troops to force Iraq to leave Kuwait?

- Q13
- https://doi.org/10.25940/ROPER-31088670

• Here are some conditions under which the United States might consider withdrawing its troops from the Mideast. For each, please tell me if you favor or oppose the U.S. withdrawing its troops if that condition is met.... (Rotate) If Iraq allows all U.S. and other foreign citizens to leave Kuwait, but Iraqi troops remain in Kuwait and Saddam Hussein remains in power?

- Q14
- https://doi.org/10.25940/ROPER-31088670

• (Here are some conditions under which the United States might consider withdrawing its troops from the Mideast. For each, please tell me if you favor or oppose the U.S. withdrawing its troops if that condition is met.)... (Rotate) If Iraq pulls all of its troops out of Kuwait, but Saddam Hussein is removed from power in Iraq

- Q16
- https://doi.org/10.25940/ROPER-31088670

• (Here are some conditions under which the United States might consider withdrawing its troops from the Mideast. For each, please tell me if you favor or oppose the U.S. withdrawing its troops if that condition is met.)... (Rotate) If Iraq pulls all of its troops out of Kuwait, but Saddam Hussein remains in power in Iraq

- Q15
- https://doi.org/10.25940/ROPER-31088670

- Just from what you have heard, read or seen, which of these statements comes closer to how you, yourself, feel about the United States presence in the Mideast? A. The United States should begin to withdraw its troops or B. The United States should continue with its present level of troop presence, or C. The United States should increase the level of its troops to force Iraq to leave Kuwait?
    - Q7
    - https://doi.org/10.25940/ROPER-31088690

- If (Saddam) Hussein pulls his troops out of all of Kuwait, should the United States keep a military presence in the Gulf to maintain stability in the region or not?
    - Q68
    - https://doi.org/10.25940/ROPER-31092941

- If (Saddam) Hussein releases the hostages and pulls his troops out of most of Kuwait, but holds on to an oilfield and some other land in Kuwait, what do you think the next step should be? Should we remove our military troops from the Mideast, or should we leave our military troops and enforce the economic embargo, but don t use military force against Iraq, or should we invade Iraq if it doesn t leave all of Kuwait?
    - Q66
    - https://doi.org/10.25940/ROPER-31092941

- As you may know, (Saddam) Hussein has ordered the release of all hostages in Iraq and Kuwait. Once the hostages have been released, what do you think the next step should be? Should we remove our military troops from the Mideast, or should we leave our military troops and enforce the economic embargo, but don t use military force against Iraq, or should we invade Iraq if they don t get out of Kuwait by the United Nations deadline of January 15 (1991)?
    - Q65
    - https://doi.org/10.25940/ROPER-31092941

- If (Saddam) Hussein releases the hostages and pulls his troops out of all of Kuwait, what do you think the next step should be? Should we remove our military troops from the Mideast, or should we leave our military troops and enforce the economic embargo, but don t use military force against Iraq, or should we invade Iraq to destroy its military threat in the region?
    - Q67
    - https://doi.org/10.25940/ROPER-31092941

- If the United States does succeed in getting Iraq to leave Kuwait--either through military or diplomatic means--do you think our troops should stay in the region indefinitely after that point to keep the peace or not?
    - Q26
    - https://doi.org/10.25940/ROPER-31088696

- Once the war in the Persian Gulf is over, do you think the United States should keep some troops in the Gulf in order to insure stability in the region, or should the United States withdraw all its troops from the area after the war?
    - Q18
    - https://doi.org/10.25940/ROPER-31105563

- Once the war in the Persian Gulf is over, do you think the United States should keep some troops in the Gulf in order to insure stability in the region, or should the United States withdraw all its troops from the area after the war?
    - Q15
    - https://doi.org/10.25940/ROPER-31091361

- In your view, what should the United States do now in Somalia:...One: Withdraw U.S. troops right away. Two: Gradually withdraw U.S. troops. Three: Keep U.S. involvement the same. Four: Increase U.S. military commitment
    - Q7

- https://doi.org/10.25940/ROPER-31088207

- What should the United States do now? Do you think the United States should keep its troops in Somalia until the situation in Somalia is peaceful, or should the United States withdraw its troops as quickly as possible?
  - Q19
  - https://doi.org/10.25940/ROPER-31090774

- How long do you think the U.S. should keep its troops in the Persian Gulf area--until Saddam Hussein leaves power, just until the current threat by Saddam Hussein is resolved, or should U.S. troops leave now?
  - Q14
  - https://doi.org/10.25940/ROPER-31088231

- Do you feel the US (United States) and its NATO (North Atlantic Treaty Organization) allies should have continued military action in Yugoslavia until Slobodan Milosevic was removed from power or not?
  - Q19
  - https://doi.org/10.25940/ROPER-31088398

- Do you think the U.S. (United States) should increase its military action in Afghanistan, keep it about the same, decrease it, or end all military action in Afghanistan?
  - Q69
  - https://doi.org/10.25940/ROPER-31092208

- Here are three statements about what the role of the US (United States) military should be in Iraq in the post war period. Please select the one that comes closest to your views....The US military should remain in Iraq, provide security and be in charge of all relief and reconstruction efforts. The US military should remain in Iraq and provide security, but the UN (United Nations) and international aid organizations should be in charge of relief and reconstruction. The US military should withdraw completely from Iraq shortly after the war is over.
  - Q28
  - https://doi.org/10.25940/ROPER-31098137

- Here are three statements about what the role of the US (United States) military should be in Iraq in the post war period. Please select the one that comes closest to your views....The US military should remain in Iraq, provide security and be in charge of all relief and reconstruction efforts. The US military should remain in Iraq and provide security, but the UN (United Nations) and international aid organizations should be in charge of relief and reconstruction. The US military should withdraw completely from Iraq.
  - Q20
  - https://doi.org/10.25940/ROPER-31098140

- Do you support or oppose the current U.S. (United States) military presence in Iraq? (If Support/Oppose, ask:) Do you support/oppose it strongly or somewhat?
  - Q8
  - https://doi.org/10.25940/ROPER-31086901

- There is some discussion about how many troops the US (United States) should have in Iraq now. Do you think the number of US troops in Iraq should be...increased a lot, increased some, maintained at the current level, decreased some, decreased a lot, or withdrawn completely?
  - Q21
  - https://doi.org/10.25940/ROPER-31098140

- Which of the following, if any, would you support in response to the attacks on U.S. (United States) military personnel by Iraqi insurgents since major combat ended? Would you support... a withdrawal of U.S. military personnel?
  - Q21
  - https://doi.org/10.25940/ROPER-31096800

- Do you think the United States should keep its military forces in Iraq until civil order is restored there, even if that means continued U.S. military casualties, or do you think the United States should withdraw its military forces from Iraq in order to avoid further U.S. military casualties, even if that means civil order is not restored there?
    - Q15
    - https://doi.org/10.25940/ROPER-31086901

- Which of the following, if any, would you support in response to the attacks on U.S. (United States) military personnel by Iraqi insurgents since major combat ended? Would you support...a withdrawal of U.S. military personnel from Iraq or not?
    - Q28
    - https://doi.org/10.25940/ROPER-31096799

- When it comes to how long American troops should stay in Iraq, which comes closer to your view?...
Statement A: American troops should be withdrawn from Iraq now or as soon as possible. Statement B: American troops should be withdrawn from Iraq according to a specified timetable and leave within the next 18 months, regardless of the situation at that time. Statement C: American troops should stay as long as necessary to complete the process, even if it takes as long as five years.
    - Q71
    - https://doi.org/10.25940/ROPER-31094827

- Do you think the United States should keep its military forces in Iraq until civil order is restored there, even if that means continued U.S. military casualties, or do you think the United States should withdraw its military forces from Iraq in order to avoid further U.S. military casualties, even if that means civil order is not restored there?
    - Q4
    - https://doi.org/10.25940/ROPER-31109492

- Which of the following steps, if any, would you support in response to the attacks on U.S. (United States) military personnel and other targets by anti-American forces in Iraq since major combat ended? Would you support...a withdrawal of U.S. military personnel or not?
    - Q22
    - https://doi.org/10.25940/ROPER-31096801

- When it comes to how long American troops should stay in Iraq, which comes closer to your view?
Statement A: American troops should be withdrawn from Iraq now or as soon as possible. Statement B: American troops should be withdrawn from Iraq according to a specified timetable and leave within the next 18 months regardless of the situation at that time. Statement C: American troops should stay as long as necessary to complete the process, even if it takes as long as five years.
    - Q51
    - https://doi.org/10.25940/ROPER-31094828

- When it comes to how long American troops should stay in Iraq, which comes closer to your view?...
Statement A: American troops should be withdrawn from Iraq now or as soon as possible. Statement B: American troops should be withdrawn from Iraq according to a specified timetable and leave within the next 18 months regardless of the situation at that time. Statement C: American troops should stay as long as necessary to complete the process, even if it takes as long as five years.
    - Q43
    - https://doi.org/10.25940/ROPER-31094829

- Which of the following statements comes closest to your view? The U.S. (United States) should keep military forces in Iraq until democracy is established there, even if that means continued U.S. casualties. The U.S. should keep military forces in Iraq until order is restored, but should not insist on establishing democracy if that means continued casualties. The U.S. should withdraw its forces from Iraq in order to avoid more casualties, even if that means order is not restored.
    - Q63
    - https://doi.org/10.25940/ROPER-31093162

- Do you think the United States should keep its military forces in Iraq until civil order is restored there, even if that means continued U.S. military casualties, or do you think the United States should withdraw its military forces from Iraq in order to avoid further U.S. military casualties, even if that means civil order is not restored there?
    - Q6
    - https://doi.org/10.25940/ROPER-31109587

- When it comes to how long American troops should stay in Iraq, which comes closer to your view?...Statement A: American troops should be withdrawn from Iraq now or as soon as possible. Statement B: American troops should be withdrawn from Iraq according to a specified timetable and leave within the next 18 months regardless of the situation at that time. Statement C: American troops should stay as long as necessary to complete the process, even if it takes as long as five years.
    - Q32
    - https://doi.org/10.25940/ROPER-31094830

- Should U.S. (United States) troops stay in Iraq as long as it takes to make sure Iraq is a stable democracy, even if that takes a long time, or should U.S. troops turn over control to Iraqis as soon as possible, even if Iraq is not completely stable?
    - Q77
    - https://doi.org/10.25940/ROPER-31091502

- When it comes to how long American troops should stay in Iraq, which comes closer to your view?...Statement A: American troops should be withdrawn from Iraq now or as soon as possible. Statement B: American troops should be withdrawn form Iraq according to a specified timetable and leave within the next 18 months regardless of the situation at that time. Statement C: American troops should stay as long as necessary to complete the process, even if it takes as long as five years.
    - Q47
    - https://doi.org/10.25940/ROPER-31094830

- Should U.S. (United States) troops stay in Iraq as long as it takes to make sure Iraq is a stable democracy, even if that takes a long time, or should U.S. troops turn over control to Iraqis as soon as possible, even if Iraq is not completely stable?
    - Q41
    - https://doi.org/10.25940/ROPER-31109618

- Do you think the United States should withdraw its military forces from Iraq now that Saddam (Hussein) has been captured, or do you think the United States should keep its military forces in Iraq until a stable government is in place there?
    - Q13
    - https://doi.org/10.25940/ROPER-31086906

- When it comes to how long American troops should stay in Iraq, which comes closer to your view?...Statement A: American troops should be withdrawn from Iraq now or as soon as possible. Statement B: American troops should be withdrawn from Iraq according to a specified timetable and leave within the next 18 months regardless of the situation at that time. Statement C: American troops should stay as long as necessary to complete the process, even if it takes as long as five years.
    - Q78
    - https://doi.org/10.25940/ROPER-31094831

- When it comes to how long American troops should stay in Iraq, which comes closer to your view?... Statement A: American troops should be withdrawn from Iraq now or as soon as possible. Statement B: American troops should be withdrawn from Iraq according to a specified timetable and leave within the next 18 months regardless of the situation at that time. Statement C: American troops should stay as long as necessary to complete the process, even if it takes as long as five years.
    - Q55
    - https://doi.org/10.25940/ROPER-31094832

- Do you think the United States should keep its military forces in Iraq until civil order is restored there, even if that means continued U.S. military casualties, or do you think the United States should withdraw its military forces from Iraq in order to avoid further U.S. military casualties, even if that means civil order is not restored there?
    - Q62
    - https://doi.org/10.25940/ROPER-31086911

- Do you think the U.S. (United States) should keep military troops in Iraq until a stable government is established there, or do you think the U.S. should bring its troops home as soon as possible?
    - Q13
    - https://doi.org/10.25940/ROPER-31095820

- Do you think the United States should keep its military forces in Iraq until civil order is restored there, even if that means continued U.S. military casualties, or do you think the United States should withdraw its military forces from Iraq in order to avoid further U.S. military casualties, even if that means civil order is not restored there?
    - Q29
    - https://doi.org/10.25940/ROPER-31086913

- Do you think the United States should keep its military forces in Iraq until civil order is restored there, even if that means continued U.S. military casualties, or do you think the United States should withdraw its military forces from Iraq in order to avoid further U.S. military casualties, even if that means civil order is not restored there?
    - Q59
    - https://doi.org/10.25940/ROPER-31086914

- Should the United States troops stay in Iraq as long as it takes to make sure Iraq is a stable democracy, even if that takes a long time, or should U.S. troops leave Iraq as soon as possible, even if Iraq is not completely stable?
    - Q26
    - https://doi.org/10.25940/ROPER-31091507

- As you may know, the number of U.S. (United States) military deaths in Iraq will soon surpass 1,000. Which comes closer to your view about what this number of deaths means for the U.S.--it is a tragic loss of life, and it is a sign that the U.S. should intensify its efforts to withdraw troops from Iraq, or it is a tragic loss of life, but it does not mean the U.S. should change its policy toward Iraq?
    - Q72
    - https://doi.org/10.25940/ROPER-31088556

- Should the United States troops stay in Iraq as long as it takes to make sure Iraq is a stable democracy, even if that takes a long time, or should U.S. troops leave Iraq as soon as possible, even if Iraq is not completely stable?
    - Q66
    - https://doi.org/10.25940/ROPER-31109840

- Do you think the United States should keep its military forces in Iraq until civil order is restored there, even if that means continued U.S. military casualties, or do you think the United States should withdraw its military forces from Iraq in order to avoid further U.S. military casualties, even if that means civil order is not restored there?
    - Q11
    - https://doi.org/10.25940/ROPER-31086920

- Should the United States troops stay in Iraq as long as it takes to make sure Iraq is a stable democracy, even if it takes a long time, or should the U.S. troops leave Iraq as soon as possible, even if Iraq is not completely stable?
    - Q71
    - https://doi.org/10.25940/ROPER-31091513

- Should the United States troops stay in Iraq as long as it takes to make sure Iraq is a stable democracy, even if it takes a long time, or should U.S. troops leave Iraq as soon as possible, even if Iraq is not completely stable?
    - Q72
    - https://doi.org/10.25940/ROPER-31091514

- Do you think the United States should keep its military forces in Iraq until civil order is restored there, even if that means continued U.S. military casualties, or do you think the United States should withdraw its military forces from Iraq in order to avoid further U.S. military casualties, even if that means civil order is not restored there?
    - Q7
    - https://doi.org/10.25940/ROPER-31086928

- Do you think the U.S. (United States) should keep military troops in Iraq until the situation has stabilized, or do you think the U.S. should bring its troops home as soon as possible?
    - Q38
    - https://doi.org/10.25940/ROPER-31095851

- Do you think the United States should keep its military forces in Iraq until civil order is restored there, even if that means continued U.S. military casualties, or do you think the United States should withdraw its military forces from Iraq in order to avoid further U.S. military casualties, even if that means civil order is not restored there?
    - Q15
    - https://doi.org/10.25940/ROPER-31086930

- As you may know, the number of U.S. (United States) military deaths in Iraq will soon surpass 2,000. Which comes closer to your view about what this number of deaths means for the U.S.--it is a tragic loss of life, and it is a sign that the U.S. should intensify its efforts to withdraw troops from Iraq, or it is a tragic loss of life, but it does not mean the U.S. should change its policy toward Iraq?
    - Q25
    - https://doi.org/10.25940/ROPER-31088625

- Which comes closest to your view about what the U.S. (United States) should now do about the number of U.S. troops in Iraq--the U.S. should send more troops to Iraq, the U.S. should keep the number of troops as it is now, the U.S. should withdraw some troops from Iraq, or the U.S. should withdraw all of its troops from Iraq?
    - Q21
    - https://doi.org/10.25940/ROPER-31088625

- Should the United States troops stay in Iraq as long as it takes to make sure Iraq is a stable democracy, even if it takes a long time, or should U.S. troops leave Iraq as soon as possible, even if Iraq is not completely stable?
    - Q40
    - https://doi.org/10.25940/ROPER-31090955

- Do you think the U.S. (United States) should keep military troops in Iraq until the situation has stabilized, or do you think the U.S. should bring its troops home as soon as possible?
    - Q52
    - https://doi.org/10.25940/ROPER-31095853

- Do you think the United States should keep its military forces in Iraq until civil order is restored there, even if that means continued U.S. military casualities, do you think the United States should withdraw its military forces from Iraq in order to avoid further U.S military causalities, even if that means civil order is not restored there?
    - Q25
    - https://doi.org/10.25940/ROPER-31086933

- Should the United States troops stay in Iraq as long as it takes to make sure Iraq is a stable democracy, even if it takes a long time, or should U.S. troops leave Iraq as soon as possible, even if Iraq is not completely stable?
    - Q46
    - https://doi.org/10.25940/ROPER-31090954

- From what you have seen or heard about the situation in Iraq, what should the United States do now--should the U.S. increase the number of U.S. troops in Iraq, keep the same number of U.S. troops in Iraq as there are now, decrease the number of U.S. troops in Iraq, or remove all its troops from Iraq?
    - Q50
    - https://doi.org/10.25940/ROPER-31091517

- Do you think the U.S. (United States) should keep military troops in Iraq until the situation has stabilized, or do you think the U.S. should bring its troops home as soon as possible?
    - Q32
    - https://doi.org/10.25940/ROPER-31095855

- President (George W.) Bush has said that withdrawing U.S. (United States) troops from Iraq now would be a recipe for disaster. Do you agree or disagree with that?
    - Q67
    - https://doi.org/10.25940/ROPER-31091517

- More specifically, do you think that we should have an immediate and orderly withdrawal of all troops from Iraq, or not?
    - Q54
    - https://doi.org/10.25940/ROPER-31094847

- If your representative in Congress called for an immediate withdrawal of U.S. (United States) troops from Iraq, would that make you more likely to vote for that representative, less likely to vote for that representative, or wouldn t it make much of a difference to you?
    - Q69
    - https://doi.org/10.25940/ROPER-31091517

- Some people say the Bush administration should set a deadline for withdrawing U.S. (United States) military forces from Iraq in order to avoid further casualties. Others say knowing when the U.S. would pull out would only encourage the anti-government insurgents. Do you yourself think the United States should or should not set a deadline for withdrawing U.S. forces from Iraq?
    - Q25
    - https://doi.org/10.25940/ROPER-31086922

- If a candidate for Congress favors setting a deadline for withdrawing U.S. (United States) forces from Iraq within the next year or so, would that make you more likely to support that candidate for Congress, more likely to oppose that candidate, or wouldn t it make much difference in your vote?
    - Q27
    - https://doi.org/10.25940/ROPER-31086922

- Which comes closest to your view about what the US (United States) should now do about the number of US troops in Iraq--the US should send more troops to Iraq, the US should keep the number of troops as it is now, the US should withdraw some troops from Iraq, or the US should withdraw all of its troops from Iraq?
    - Q62
    - https://doi.org/10.25940/ROPER-31088635

- Should the United States troops stay in Iraq as long as it takes to make sure Iraq is a stable democracy, even if it takes a long time, or should U.S. troops leave Iraq as soon as possible, even if Iraq is not completely stable?
    - Q54
    - https://doi.org/10.25940/ROPER-31090957

- Should the United States troops stay in Iraq as long as it takes to make sure Iraq is a stable democracy, even if it takes a long time, or should U.S. troops leave Iraq as soon as possible, even if Iraq is not completely stable?
    - Q57
    - https://doi.org/10.25940/ROPER-31091518

- Do you think the U.S. (United States) should keep military troops in Iraq until the situation has stabilized, or do you think the U.S. should bring its troops home as soon as possible?
    - Q22
    - https://doi.org/10.25940/ROPER-31095869

- (I m going to read you several characteristics of a possible candidate for Congress. For each one I mention, please tell me whether you would be more likely or less likely to vote for a candidate for Congress with that characteristic, or whether it would make no difference in your vote either way.) A candidate who...favors withdrawing all American troops from Iraq in the next twelve months.
    - Q21
    - https://doi.org/10.25940/ROPER-31094849

- Do you think the U.S. (United States) should keep military troops in Iraq until the situation has stabilized, or do you think the U.S. should bring its troops home as soon as possible?
    - Q46
    - https://doi.org/10.25940/ROPER-31095871

- More specifically, do you think that we should have an immediate and orderly withdrawal of all troops from Iraq, or not?
    - Q60
    - https://doi.org/10.25940/ROPER-31094849

- (Let me read you a number of proposals people have made for ways in which America could reduce its commitments and military forces in Iraq. For each one, please tell me whether that is something you would definitely support, something you think should be considered, or something you would definitely oppose.)...Give Iraqis specific deadlines for recruiting and training security forces, and withdraw U.S. (United States) troops on the basis of this, even if the Iraqis fall short of their recruitment and training goals.
    - Q74
    - https://doi.org/10.25940/ROPER-31094849

- (I m going to read you several characteristics of a possible candidate for Congress. For each one I mention, please tell me whether you would be more likely or less likely to vote for a candidate for Congress with that characteristic, or whether it would make no difference in your vote either way.) A candidate who...favors staying in Iraq as long as is necessary to create a stable Iraqi democracy.
    - Q25
    - https://doi.org/10.25940/ROPER-31094849

- Whether you think Iraq is in a civil war or not, if the sectarian violence there were to turn into a nationwide civil war, what do you think the United States should do: should the U.S. choose a side and fight with that group, or should the U.S. keep troops in Iraq but remain neutral and mediate a cease fire, or should the U.S. withdraw its troops, or should the U.S. intervene to stop the fighting?
    - Q51
    - https://doi.org/10.25940/ROPER-31093192

- Should the United States troops stay in Iraq as long as it takes to make sure Iraq is a stable democracy, even if it takes a long time, or should US troops leave Iraq as soon as possible, even if Iraq is not completely stable?
    - Q37
    - https://doi.org/10.25940/ROPER-31090960

- From what you have seen or heard about the situation in Iraq, what should the United States do now--should the US increase the number of US troops in Iraq, keep the same number of US troops in Iraq as there are now, decrease the number of US troops in Iraq, or remove all its troops from Iraq?
    - Q39
    - https://doi.org/10.25940/ROPER-31090960

- Do you think the U.S. (United States) should keep military troops in Iraq until the situation has stabilized, or do you think the U.S. should bring its troops home as soon as possible?
    - Q69
    - https://doi.org/10.25940/ROPER-31095872

- Some people say the Bush administration should set a deadline for withdrawing the US (United States) military forces from Iraq in order to avoid further casualties. Others say knowing when the US would pull out would only encourage the anti-government insurgents. Do you yourself think the United States should or should not set a deadline for withdrawing US forces from Iraq?
    - Q23
    - https://doi.org/10.25940/ROPER-31086939

- Here are four different plans the US (United States) could follow in dealing with the war in Iraq. Which one do you prefer--withdraw all troops from Iraq immediately, withdraw all troops by July 2007--that is, in 12 months time, withdraw troops, but take as many years to do this as are needed to turn control over to the Iraqis, or send more troops to Iraq?
    - Q10
    - https://doi.org/10.25940/ROPER-31089646

- Should the United States troops stay in Iraq as long as it takes to make sure Iraq is a stable democracy, even if it takes a long time, or should US troops leave Iraq as soon as possible, even if Iraq is not completely stable?
    - Q16
    - https://doi.org/10.25940/ROPER-31090964

- More specifically, do you think that we should have an immediate and orderly withdrawal of all troops from Iraq, or not?
    - Q64
    - https://doi.org/10.25940/ROPER-31094852

- From what you have seen or heard about the situation in Iraq, what should the United States do now--should the US increase the number of US troops in Iraq, keep the same number of US troops in Iraq as there are now, decrease the number of US troops in Iraq, or remove all its troops from Iraq?
    - Q32
    - https://doi.org/10.25940/ROPER-31091525

- Do you think the US (United States) should keep military troops in Iraq until the situation has been stabilized, or do you think the US should bring its troops home as soon as possible?
    - Q53
    - https://doi.org/10.25940/ROPER-31095892

- (Please tell me if you support or oppose each of the following proposals.) How about...withdrawing almost all US (United States) combat forces from Iraq by early 2008, but keeping military training forces there?
    - Q28
    - https://doi.org/10.25940/ROPER-31086944

- Which comes closest to your view--the US (United States) should continue fighting the war in Iraq using the same military strategy and tactics it is using now, the US should continue fighting the war in Iraq, but needs to change its strategy and tactics, or the US should take steps to end its

involvement in Iraq?
- Q28
- https://doi.org/10.25940/ROPER-31090966

• Do you think the United States should keep its military forces in Iraq until civil order is restored there, even if that means continued US military casualties, or do you think the United States should withdraw its military forces from Iraq in order to avoid further US military casualties, even if that means civil order is not restored there?
- Q15
- https://doi.org/10.25940/ROPER-31086944

• Here are four different plans the US (United States) could follow in dealing with the war in Iraq. Which one do you prefer--withdraw all troops from Iraq immediately, withdraw all troops by December, 2007--that is, in 12 months time--withdraw troops, but take as many years to do this as are needed to turn control over to the Iraqis, or send more troops to Iraq?
- Q3
- https://doi.org/10.25940/ROPER-31095409

• As you may know, Congress has authorized 70 billion dollars for the war in Iraq. Would you favor or oppose a proposal that would spend all of that money to immediately withdraw all US (United States) troops from Iraq and would prevent the US from spending any additional money for future military operations in that country?
- Q12
- https://doi.org/10.25940/ROPER-31095409

• Which of the following three alternatives would you most like to see the American military in Iraq take? A) Continue working toward establishing a secure Iraqi government using American troops in all aspects of the war. B) Re-deploy American troops so that they only train and support Iraqi forces, but do not leave the secure American bases. C) Have a phased withdrawal of American troops beginning immediately.
- Q70
- https://doi.org/10.25940/ROPER-31094857

• From what you have seen or heard about the situation in Iraq, what should the United States do now--should the US increase the number of US troops in Iraq, keep the same number of US troops in Iraq as there are now, decrease the number of US troops in Iraq, or remove all of its troops from Iraq?
- Q19
- https://doi.org/10.25940/ROPER-31090966

• (Do you think the Bush administration should or should not follow each of these recommendations made by the Iraq Study Group?) How about...withdraw most US combat troops from Iraq by March 2008, leaving only a limited number to help train and advise the Iraqis?
- Q52
- https://doi.org/10.25940/ROPER-31089663

• Do you think the US (United States) should keep military troops in Iraq until the situation has stabilized, or do you think the US should bring its troops home as soon as possible?
- Q13
- https://doi.org/10.25940/ROPER-31095876

• Which comes closest to your view?...The US (United States) should continue fighting the war in Iraq using the same military strategy and tactics it is using now. The US should continue fighting the war in Iraq, but needs to change its strategy and tactics. The US should take steps to end its involvement in Iraq.
- Q48
- https://doi.org/10.25940/ROPER-31090967

• In your opinion, should the United States withdraw troops from Iraq right away, or should the US

begin bringing troops home within the next year, or should troops stay in Iraq for as long as it takes to win the war?

- Q9
- https://doi.org/10.25940/ROPER-31093203

• I m going to read you several possible outcomes to the war in Iraq. Please tell me which one of these would be the most acceptable outcome to you....A) There is an increase in the number of troops now, and US (United States) troops leave only after Iraq becomes a stable democracy, however long this takes. B) US troops leave within the next year even if violence in Iraq continues, but some troops remain in the region to prevent the conflict from spreading. C) US troops begin the process of leaving now regardless of conditions in Iraq. Or are none of these acceptable to do?

- Q62
- https://doi.org/10.25940/ROPER-31094858

• Do you support or oppose the idea of withdrawing most US (United States) troops from Iraq by early 2008, while keeping military training forces there to assist and train Iraqi troops? (If Support/Oppose, ask:) Do you support/oppose this strongly, or only somewhat?

- Q10
- https://doi.org/10.25940/ROPER-31093203

• (Congress may consider several different resolutions on President (George W.) Bush s plan to send more troops to Iraq. Some resolutions would take steps to prevent this, while other resolutions would express opposition to the plan, but not attempt to block it.)...And thinking about the US (United States) troops currently stationed in Iraq, what would you want your members of Congress to do--should they vote to allow the government to spend money to keep those troops in that country, or vote to allow the government to spend money only to withdraw those troops from Iraq?

- Q26
- https://doi.org/10.25940/ROPER-31095410

• From what you have seen or heard about the situation in Iraq, what should the United States do now--should the US increase the number of US troops in Iraq, keep the same number of US troops in Iraq as there are now, decrease the number of US troops in Iraq, or remove all its troops from Iraq?

- Q43
- https://doi.org/10.25940/ROPER-31090967

• Do you think the United States should keep its military forces in Iraq until civil order is restored there, even if that means continued US military casualties, or do you think the United States should withdraw its military forces from Iraq in order to avoid further US military casualties, even if that means civil order is not restored there?

- Q35
- https://doi.org/10.25940/ROPER-31086946

• Do you think the US (United States) should keep military troops in Iraq until the situation has stabilized, or do you think the US should bring its troops home as soon as possible?

- Q22
- https://doi.org/10.25940/ROPER-31095898

• Do you support or oppose the legislation passed this week by the US (United States) Senate calling for the withdrawal of US troops from Iraq by March 2008?

- Q6
- https://doi.org/10.25940/ROPER-31096891

• As you may know, Democrats have proposed various ways of ending the war in Iraq. One of them is a proposal to withdraw all combat brigades from Iraq by March 31, 2008. Do you support or oppose this proposal? (If Support/Oppose, ask:) Do you support/oppose the idea strongly, or only somewhat?

- Q50
- https://doi.org/10.25940/ROPER-31093206

- The Congress is now debating future funding for the war in Iraq. Would you like to see your Congressional representative vote for or against a bill that calls for a withdrawal of troops from Iraq to be completed by August of 2008?
    - Q4
    - https://doi.org/10.25940/ROPER-31095899

- Do you think the US (United States) should keep military troops in Iraq until the situation has stabilized, or do you think the US should bring its troops home as soon as possible?
    - Q9
    - https://doi.org/10.25940/ROPER-31095899

- Which one of the following plans for US (United States) policy in Iraq comes closest to your own position?...Withdraw all troops from Iraq in the next 12 months or so whatever happens in Iraq., Set sliding deadlines for withdrawing most US combat troops by August 31, 2008, with the withdrawal schedule depending on the Iraqi government s progress in training its forces and bringing stability to Iraq. Keep US troops in Iraq as long as needed until the Iraqis can handle the situation themselves.
    - Q6
    - https://doi.org/10.25940/ROPER-31097920

- Do you favor or oppose Congressional legislation that would require the withdrawal of US (United States) troops from Iraq by the fall of 2008?
    - Q8
    - https://doi.org/10.25940/ROPER-31096890

- Do you think the US (United States) should keep military troops in Iraq until the situation has stabilized, or do you think the US should bring its troops home as soon as possible?
    - Q62
    - https://doi.org/10.25940/ROPER-31095900

- If you had to choose, which would you favor--the U.S. (United States) setting a timetable for removing its troops from Iraq and sticking to that timetable regardless of what is happening in Iraq, or the U.S. keeping troops in Iraq as long as necessary to secure the country, even if that takes many more years?
    - Q18
    - https://doi.org/10.25940/ROPER-31110889

- The Congress is now debating future funding for the war in Iraq. Would you like to see your Congressional representative vote for or against a bill that calls for a withdrawal of troops from Iraq to be completed by August of 2008?
    - Q46
    - https://doi.org/10.25940/ROPER-31095900

- Do you think the US (United States) should keep military troops in Iraq until the situation has stabilized, or do you think the US should bring its troops home as soon as possible?
    - Q64
    - https://doi.org/10.25940/ROPER-31095901

- One proposal would provide additional funds for US (United States) troops in Iraq and would require the US to start withdrawing all its troops from Iraq by a specific date. Would you favor or oppose this bill?
    - Q15
    - https://doi.org/10.25940/ROPER-31095415

- One proposal would not provide additional funds for US (United States) troops in Iraq and would require the US to withdraw all its troops by March, 2008. Would you favor or oppose this bill?
    - Q17
    - https://doi.org/10.25940/ROPER-31095415

- In your opinion, should the United States withdraw troops from Iraq right away, or should the US begin bringing troops home within the next year, or should the troops stay in Iraq for as long as it takes to win the war?
    - Q42
    - https://doi.org/10.25940/ROPER-31093208

- From what you have seen or heard about the situation in Iraq, what should the United States do now--should the US increase the number of US troops in Iraq, keep the same number of US troops in Iraq as there are now, decrease the number of US troops in Iraq, or remove all its troops from Iraq?
    - Q30
    - https://doi.org/10.25940/ROPER-31090975

- Which of the following four choices comes closest to your view about what the US (United States) should do in Iraq?...Begin an immediate withdrawal of all US troops in Iraq. Start withdrawing troops by the fall, with all troops out by next spring. Leave a substantial number of troops in Iraq, but have them fall back to their bases and concentrate on training. Make no cutbacks in US troops.
    - Q4
    - https://doi.org/10.25940/ROPER-31096896

- From what you have seen or heard about the situation in Iraq, what should the United States do now--should the US increase the number of US troops in Iraq, keep the same number of US troops in Iraq as there are now, decrease the number of troops in Iraq, or remove all its troops from Iraq?
    - Q10
    - https://doi.org/10.25940/ROPER-31091534

- Later this summer (2007), Congress will be debating future funding for the war in Iraq. If a bill is proposed that calls for a withdrawal of troops from Iraq to be completed by next year, would you like to see your Congressional representative vote for or against it?
    - Q29
    - https://doi.org/10.25940/ROPER-31095902

- Do you think the US (United States) should keep military troops in Iraq until the situation has stabilized, or do you think the US (United States) should bring its troops home as soon as possible?
    - Q38
    - https://doi.org/10.25940/ROPER-31095902

- From what you know about the US (United States) involvement in Iraq, how much longer would you be willing to have large numbers of US troops remain in Iraq--less than a year, one to two years, two to five years or longer than five years?
    - Q29
    - https://doi.org/10.25940/ROPER-31090977

- Looking ahead in Iraq, if you had to choose, which of these comes closest to your position?...The United States should withdraw all of its troops within the next year regardless of what happens in Iraq after the troops leave. The United States should withdraw some troops but leave some troops to train Iraqi forces, conduct raids against terrorist groups and protect American diplomats. The United States should keep the same number of troops in Iraq as are there now and continue to fight until there is a stable democracy in Iraq.
    - Q60
    - https://doi.org/10.25940/ROPER-31090977

- Do you think the US (United States) should keep military troops in Iraq until the situation has stabilized, or do you think the US should bring its troops home as soon as possible?
    - Q5
    - https://doi.org/10.25940/ROPER-31095904

- Looking ahead in Iraq, if you had to choose, which of these comes closest to your position?...The United States should withdraw all of its troops within the next year regardless of what happens in Iraq after the troops leave. The United States should withdraw some troops but leave some troops to train Iraqi forces and protect American diplomats. The United States should keep the same number of troops in Iraq as there are now and continue to fight until there is a stable democracy in Iraq.
    - Q61
    - https://doi.org/10.25940/ROPER-31090977

- From what you have seen or heard about the situation in Iraq, what should the United States do now--should the US increase the number of US troops in Iraq, keep the same number of US troops in Iraq as there are now, decrease the number of troops in Iraq, or remove all its troops from Iraq?
    - Q32
    - https://doi.org/10.25940/ROPER-31090977

- Currently, there are three different interpretations of the recent troop surge and its value in achieving victory in Iraq. Please tell me which comes closest to your point of view....A: Continuing in the same direction and keeping the troops in Iraq makes sense, as long as military progress is being made. B: Continuing in the same direction and keeping troops in Iraq only makes sense if there is progress on Iraq s political and governmental situation. C: Continuing in the same direction and keeping the troops in Iraq does not make sense, regardless of any military or political progress.
    - Q75
    - https://doi.org/10.25940/ROPER-31094862

- I m going to read you several possible outcomes to the war in Iraq. Please tell me which one of these would be the most acceptable outcome to you....A: Maintain the number of troops there now, and US (United States) troops leave only after Iraq becomes a stable democracy, however long this takes. B: US troops leave within the next year even if violence in Iraq continues, but some troops remain in the region to prevent the conflict from spreading. C: US troops begin the process of leaving now regardless of conditions in Iraq. Or, are none of these acceptable to do?
    - Q81
    - https://doi.org/10.25940/ROPER-31094862

- Which comes closest to your view about what the US (United States) should now do about the number of US troops in Iraq--the US should send more troops to Iraq, the US should keep the number of troops as it is now, the US should withdraw some troops from Iraq, or the US should withdraw all of its troops from Iraq?
    - Q5
    - https://doi.org/10.25940/ROPER-31095418

- From what you know about the US (United States) involvement in Iraq, how much longer would you be willing to have large numbers of US troops remain in Iraq--less than a year, one to two years, two to five years or longer than five years?
    - Q13
    - https://doi.org/10.25940/ROPER-31090978

- How much longer do you think large numbers of US (United States) troops will have to remain in Iraq--less than a year, one to two years, two to five years or longer than five years?
    - Q14
    - https://doi.org/10.25940/ROPER-31090978

- Suppose you were asked to give your opinion to President (George W.) Bush and Congress about how long to keep US (United States) troops in Iraq. What would your advice be?
    - Q12
    - https://doi.org/10.25940/ROPER-31111083

- (Does each of the following make you more likely or less likely to support a (2008) candidate for president?...Much more likely, somewhat more likely, somewhat less likely, much less likely, makes no difference)...The candidate supports immediate withdrawal of troops from Iraq.

- Q126
- https://doi.org/10.25940/ROPER-31111097

• (Does each of the following make you more likely or less likely to support a (2008) candidate for president...Much more likely, somewhat more likely, somewhat less likely, much less likely, makes no difference)...The candidate supports immediate withdrawal of troops from Iraq.
  - Q292
  - https://doi.org/10.25940/ROPER-31111097

• Do you think the US (United States) should keep military troops in Iraq until the situation has stabilized, or do you think the US should bring its troops home as soon as possible?
  - Q71
  - https://doi.org/10.25940/ROPER-31095927

• From what you know about the US (United States) involvement in Iraq, how much longer would you be willing to have large numbers of US troops remain in Iraq--less than a year, one to two years, two to five years, five to ten years, or as long as it takes?
  - Q34
  - https://doi.org/10.25940/ROPER-31090987

• Do you think the United States should keep its military forces in Iraq until civil order is restored there, even if that means continued US military casualties, or do you think the United States should withdraw its military forces from Iraq in order to avoid further US military casualties, even if that means civil order is not restored there?
  - Q17
  - https://doi.org/10.25940/ROPER-31086961

• In you opinion, should the United States withdraw most of its combat troops from Iraq right away, should the US bring most of its troops home within the next two years, or should it leave combat troops in Iraq for as long as it takes to establish a more stable and secure Iraq?
  - Q83
  - https://doi.org/10.25940/ROPER-31094144

• Regarding the war in Iraq, President (Barack) Obama has announced that US (United States) forces will remain in Iraq for 18 months, and then a withdrawal will begin to bring US troops home and transfer authority to Iraq. Do you support or oppose this policy?...Strongly support, somewhat support, somewhat oppose, strongly oppose
  - Q13
  - https://doi.org/10.25940/ROPER-31112417

• (Do you approve, disapprove, or neither approve nor disapprove of Barack Obama s decision to end the combat role of US (United States) troops and remove most but not all US troops from Iraq by August 31, 2010?) (If Approve, ask:) Is that strongly approve or somewhat approve? (If Disapprove, ask:) Is that strongly disapprove or somewhat disapprove? (If Don t know/Refused, ask:) If you had to choose, do you lean more toward approving or disapproving of Barack Obama s decision to end the combat role of US troops and remove most but not all US troops from Iraq by August 31, 2010?
  - Q48
  - https://doi.org/10.25940/ROPER-31111762

• From what you know about the US (United States) involvement in Afghanistan, how much longer would you be willing to have large numbers of US troops remain in Afghanistan--less than a year, one to two years, two to five years, five to ten years, or as long as it takes?
  - Q26
  - https://doi.org/10.25940/ROPER-31091554

• Do you think the US (United States) is doing the right thing by fighting the war in Afghanistan now, or should the US not be involved in Afghanistan now?
  - Q11
  - https://doi.org/10.25940/ROPER-31091554

- President (Barack) Obama also announced that he plans to start removing US (United States) troops from Afghanistan in the summer of 2011. Thinking specifically about that policy and not about Obama s decision to announce it at this time, do you favor or oppose Obama s plan to start removing troops from Afghanistan in 2011?
    - Q5
    - https://doi.org/10.25940/ROPER-31095462

- If you had to choose, which do you think is better for the US (United States)--to keep a significant number of troops in Afghanistan until the situation there gets better, even if that takes many years, or to set a time-table for removing troops from Afghanistan and to stick to that time-table regardless of what is going on in Afghanistan at the time?
    - Q10
    - https://doi.org/10.25940/ROPER-31089758

- Which of the following do you think is the best policy for US (United States) involvement in Afghanistan?...Stick to the plan to start withdrawal of forces in July of next year (2011), even if the country is still as unstable as it is today, be open to keeping current numbers of forces in Afghanistan--or even adding more--if the country is still unstable in July of next year
    - Q28
    - https://doi.org/10.25940/ROPER-31112177

- Do you approve, disapprove or neither approve nor disapprove of Barack Obama s decision to end the combat role of US (United States) troops and remove most but not all US troops from Iraq by August 31, 2010? (If Approve, ask:) Is that strongly approve or somewhat approve? (If Disapprove, ask:) Is that strongly disapprove or somewhat disapprove? (If Don t know/Refused, ask:) If you had to choose, do you lean more toward approving or disapproving of Barack Obama s decision to end the combat role of US troops and remove most but not all of US troops from Iraq by August 31, 2010?
    - Q109
    - https://doi.org/10.25940/ROPER-31112209

- As you may know, President (Barack) Obama plans to complete withdrawal of US (United States) combat troops from Afghanistan by the end of the year 2014. Which comes closest to your view--you agree with the timetable of removing US combat troops from Afghanistan by the end of 2014, you think the US should complete withdrawing combat troops from Afghanistan before the end of 2014, or you think the US should not set a timetable and keep combat troops in Afghanistan as long as needed to achieve its objectives there?
    - Q9
    - https://doi.org/10.25940/ROPER-31089760

- Do you think the US (United States) is doing the right thing by fighting the war in Afghanistan now, or should the US not be involved in Afghanistan right now?
    - Q21
    - https://doi.org/10.25940/ROPER-31091028

- Now, we have a question about the military campaign being conducted in Libya by the United States and other countries. Do you think the United States should --take the leading role in this campaign, take a major role, but not the leading role, take a minor role, or withdraw from this campaign entirely?
    - Q4
    - https://doi.org/10.25940/ROPER-31088932

- (Do you approve, disapprove or neither approve nor disapprove of Barack Obama s decision to begin withdrawal of US (United States) combat troops from Afghanistan in July, 2011 and end US combat operations there by 2014?) (If Approve, ask:) Is that strongly approve or somewhat approve? (If Disapprove, ask:) Is that strongly disapprove or somewhat disapprove? (If Don t know/Refused, ask:) If you had to choose, do you lean more toward approving or disapproving of Barack Obama s decision to begin withdrawal of US combat troops from Afghanistan in July, 2011 and end US combat operations there by 2014?

- Q70
- https://doi.org/10.25940/ROPER-31112439

• Do you think the United States should keep its military forces in Afghanistan until it has trained the Afghan army to be self-sufficient, or do you think the United States should withdraw its military forces even if the Afghan army is not adequately trained?
- Q4
- https://doi.org/10.25940/ROPER-31087000

• As you may know, the US (United States) plans to withdraw all of its troops from Afghanistan by the end of the year 2014. Which would you prefer to see happen--for the US to stick to its timetable for withdrawing troops by 2014, speed up its withdrawal from Afghanistan, or keep troops in Afghanistan as long as it takes to accomplish its goals?
- Q1
- https://doi.org/10.25940/ROPER-31089832

• As you may know, (Barack) Obama has halted the withdrawal of US forces from Afghanistan, saying the current force of 98-hundred troops will remain in place through most of next year, and 55-hundred will stay into 2017. Is this something you support or oppose?
- Q2
- https://doi.org/10.25940/ROPER-31087031

• Overall, do you think that withdrawing US troops from Syria would be the right decision or wrong decision?
- Q12
- https://doi.org/10.25940/ROPER-31115692

• Do you think that we should have a rapid and orderly withdrawal of all troops from Afghanistan, or not?
- Q33
- https://doi.org/10.25940/ROPER-31116129

• Do you think that we should have a rapid and orderly withdrawal of all troops from Syria, or not?
- Q52
- https://doi.org/10.25940/ROPER-31116129

• As you may be aware, President (Joe) Biden is planning to withdraw all United States troops from Afghanistan by September 11 (2021). To what extent do you support or oppose this plan?...Strongly support, somewhat support, somewhat oppose, strongly oppose
- Q1
- https://doi.org/10.25940/ROPER-31118528

• Please indicate the extent to which you agree or disagree with each of the following statements....Strongly agree, somewhat agree, somewhat disagree, strongly disagree...The United States should bring home all of its troops from Afghanistan immediately.
- Q13
- https://doi.org/10.25940/ROPER-31118528

• As you may know, President (Joe) Biden has decided to withdraw all United States troops from Afghanistan by September 11, 2021. Do you approve or disapprove of President Biden s decision?
- Q36
- https://doi.org/10.25940/ROPER-31118441

• Do you think the United States decision to withdraw all troops from Afghanistan was the right decision or wrong decision?
- Q2
- https://doi.org/10.25940/ROPER-31118591

• Regarding the United States role in Afghanistan, do you think the US should have withdrawn all of

its troops, withdrawn some, but left troops in the country, not withdrawn any troops at all, or not withdrawn and sent more troops?

- Q8
- https://doi.org/10.25940/ROPER-31118602

• Which of these comes closest to your opinion regarding the withdrawal of all United States forces from Afghanistan? Would you say you support the withdrawal and approve of how (Joe) Biden has handled it, support the withdrawal but disapprove of how Biden has handled it or oppose the withdrawal of all US forces from Afghanistan?

- Q4
- https://doi.org/10.25940/ROPER-31118604

• Which comes closer to your view?...The United States has a duty to continue its involvement in Afghanistan, Afghanistan must determine its future without United States involvement

- Q7
- https://doi.org/10.25940/ROPER-31118602

• Regardless of how it was handled, do you approve or disapprove of the decision to withdraw the United States presence in Afghanistan?

- Q27
- https://doi.org/10.25940/ROPER-31118618

• Do you support or oppose the withdrawal of all United States troops from Afghanistan?

- Q7
- https://doi.org/10.25940/ROPER-31118675

• Do you approve or disapprove of...President (Joe) Biden s decision to withdraw American troops from Afghanistan? ...Strongly approve, somewhat approve, somewhat disapprove, strongly disapprove

- Q2
- https://doi.org/10.25940/ROPER-31118794

• As you may know, President (Joe) Biden decided to withdraw all United States troops from Afghanistan. Do you approve or disapprove of President Biden s decision?

- Q12
- https://doi.org/10.25940/ROPER-31118613

• Do you think that the United States did the right thing by withdrawing all troops from Afghanistan, do you think that the US should have withdrawn some troops from Afghanistan but not all troops, or do you think that the United States should not have withdrawn any troops from Afghanistan?

- Q29
- https://doi.org/10.25940/ROPER-31118703
